# A simple strategy for heritable chromosomal deletions in zebrafish via the combinatorial action of targeting nucleases

**DOI:** 10.1186/gb-2013-14-7-r69

**Published:** 2013-07-01

**Authors:** Shimin Lim, Yin Wang, Xueyao Yu, Yian Huang, Mark S Featherstone, Karuna Sampath

**Affiliations:** 1 School of Biological Sciences, 50 Nanyang Avenue, Nanyang Technological University, Singapore 639798; 2 Temasek Life Sciences Laboratory, 1 Research Link, National University of Singapore, Singapore 117604; 3 Department of Biological Sciences, National University of Singapore, 14 Science Drive 4, Singapore 117543; 4 School of Applied Science, Temasek Polytechnic, 21 Tampines Avenue 1, Singapore 529757; 5 NUS High School of Mathematics and Science, 20 Clementi Avenue 1, Singapore 129957

## Abstract

Precise and effective genome-editing tools are essential for functional genomics and gene therapy. Targeting nucleases have been successfully used to edit genomes. However, whole-locus or element-specific deletions abolishing transcript expression have not previously been reported. Here, we show heritable targeting of locus-specific deletions in the zebrafish nodal-related genes *squint *(*sqt*) and *cyclops *(*cyc*). Our strategy of heritable chromosomal editing can be used for disease modeling, analyzing gene clusters, regulatory regions, and determining the functions of non-coding RNAs in genomes.

## Background

Genome editing tools such as transcription activator-like effector nucleases (TALENs) and zinc finger nucleases (ZFNs) have revolutionized the fields of biotechnology, gene therapy and functional genomic studies in many organisms [1-4]. However, engineering large chromosomal deletions in vertebrates has been largely restricted to mice, where the typical strategy used is gene targeting, and subsequently, specific regions are engineered by site-specific recombination systems such as the Cre/Lox or Flp/FRT systems [5]. Although site-specific recombination has been used successfully to analyze the functions of genes involved in embryonic development, cancer and other diseases, this strategy is time, labor and resource intensive. Hence, rapid and facile methods to engineer chromosomes are of immense value.

Analysis of regulatory elements in the genome, and determining the activity and functions of gene clusters require generation of chromosomal lesions. Although large chromosomal lesions have been generated by gamma ray treatment and other methods, these lesions are often accompanied by complex rearrangements affecting several loci, which is a limitation for precise analysis of specific genomic regions or regulatory elements. In addition, the size and position of the re-arrangements cannot be predetermined by these methods [6,7]. Therefore, precise and easy techniques to generate segmental mutations at desired locations on chromosomes would be useful for analysis of gene clusters and large regulatory regions in the genome.

Recent genome-wide transcriptome analyses in cells and organisms have identified several non-coding and novel coding RNAs. However, determining their functions requires the generation of RNA-null alleles [8]. The TALEN and ZFN technologies have been used successfully in many organisms to generate small insertion and deletion mutations at target sites of specific genes [3,9-11]. Large chromosomal deletions and inversions have been shown in cell lines using ZFNs, and deletions using two pairs of TALENs have been generated in silkworm, swine fibroblasts and zebrafish [12-16]. So far, however, heritable chromosomal deletions that specifically abolish expression of a transcript have not been reported with these nucleases in any organism. Thus, rapid and easy methods to generate whole-locus, element-specific or transcript-specific deletions would greatly facilitate functional genomic studies.

Here, we report a simple, effective and rapid strategy to generate a whole locus deletion in zebrafish, by the simultaneous use of two pairs of TALENs or TALEN pairs in conjunction with ZFN pairs, that we used successfully to precisely delete the nodal-related gene *sqt *and generate *sqt *RNA-null alleles. We also report targeted deletions in a second zebrafish nodal gene, *cyclops *(*cyc*), for which gamma ray- and chemically induced chromosomal rearrangements and point mutations were reported, but a precise locus-specific deletion was not available [6,17-23]. Our strategy of heritable chromosomal editing can potentially be applied for functional genomic studies in a variety of organisms.

## Results

**Figure 1:**
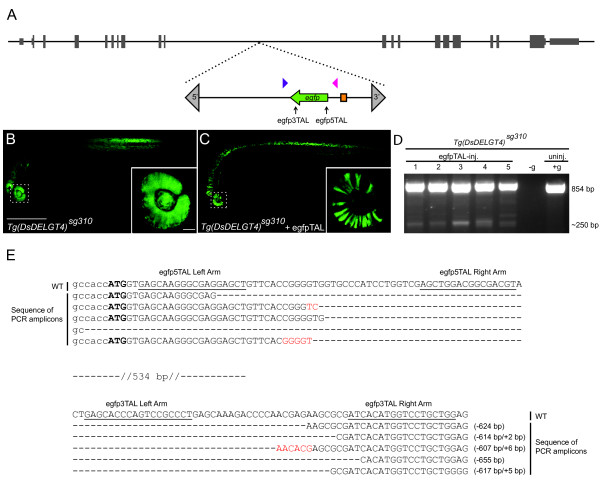
**Targeted deletions in *egfp***. **(a) **Schematic representation of chromosome 21 showing the position of the *Tg (Ds DELGT4) ^sg310 ^*enhancer trap insertion. The Ds transposon terminal repeat sequences are indicated by grey triangles; green arrow indicates *egfp *reporter sequences and orientation, orange box indicates the *glial fibrillary acidic protein *mini-promoter; TALEN targeting sites are shown with black arrows and genotyping primers are indicated by blue and magenta triangles. **(b) **A 30 hours post-fertilization (hpf) *Tg (Ds DELGT4) ^sg310 ^*embryo showing ubiquitous and robust expression of EGFP; inset shows uniform EGFP expression in the eye; scale bars, 500 μm in (b); 50 μm in inset. **(c) ***Tg (Ds DELGT4) ^sg310 ^*embryo injected with *egfp *TALEN pairs showing patchy and reduced EGFP expression; inset shows loss of EGFP expression in some sectors of the eye. **(d) **PCR with primers spanning the TALEN targeting sites (blue and magenta triangles in (a)) shows the expected 250 bp truncated *egfp *and 854 bp full-length *egfp *products in individual embryos injected with *egfp *TALEN pairs, whereas only the full-length product is observed in the un-injected control embryo. No template control is indicated by -g. **(e) **Alignment of wild-type (WT) *egfp *sequences with mutated PCR amplicons shows various deletions of approximately 600 bp between the targeting sites, accompanied by small insertions (red).

To test if large deletions can be generated efficiently by using multiple TALENs, we first targeted the reporter gene encoding enhanced green fluorescent protein (EGFP). We designed and synthesized two TALEN pairs spaced approximately 600 bp apart to target *egfp *sequences [9,24] (black arrows in Figure 1a). Each TALEN pair was tested individually and in combinations, at various doses, by injecting transgenic zebrafish embryos (*Tg (Ds DELGT4) ^sg310^*) with ubiquitous and robust EGFP expression (Figure 1b). Injected embryos were assessed for abnormalities or lethality, and for EGFP expression. The cutting efficiency of each TALEN pair was estimated by T7 endonuclease I (T7E1) assay on ten individual injected embryos (Table 1) and calculated by sequencing pooled PCR amplicons from the embryos (Figure S1A,D,H in Additional file 1). Higher doses of *egfp *TALEN pairs resulted in increased numbers of abnormal embryos and lethality (Table 2). Loss of EGFP expression was observed at 30 hours post-fertilization (hpf) in sectors of the eyes and neural tube of *egfp *TALEN-injected embryos (Figure 1c). Thus, the injected *egfp *TALEN pairs induce mutations in *egfp *and effectively disrupt EGFP expression in embryos.

**Table 1:** Mutation frequencies induced by single TALEN pairs

		Mutation frequency by T7E1 in individual embryos										
		
TALEN pair	Dosage	1	2	3	4	5	6	7	8	9	10	Mean ± SEM
egfp5TAL	12.5 pg	15.07%	8.67%	25.40%	16.99%	16.40%	20.68%	16.57%	18.42%	13.85%	9.88%	16.2 ± 1.5%
egfp3TAL	12.5 pg	25.57%	14.43%	46.31%	20.71%	30.76%	24.42%	21.81%	24.62%	11.36%	34.57%	25.5 ± 3.2%
sqt5TAL	25 pg	8.09%	7.17%	7.32%	4.18%	11.85%	8.47%	6.81%	12.83%	10.42%	13.84%	9.1 ± 1.0%
sqt3TAL	25 pg	9.77%	5.91%	4.89%	1.90%	3.46%	2.00%	4.37%	4.44%	5.48%	3.22%	4.5 ± 0.7%
cyc5TAL	12.5 pg	17.53%	46.86%	25.65%	24.72%	40.45%	27.34%	44.29%	32.57%	25.27%	34.58%	31.9 ± 3.0%
cyc3TAL	12.5 pg	43.76%	58.13%	59.00%	52.41%	57.46%	70.59%	65.23%	40.71%	41.66%	50.57%	54.0 ± 3.2%

For each single TALEN pair, ten injected embryos were assessed for mutation frequency by the T7E1 assay. SEM, standard error of the mean.

**Table 2:** Frequency of deformities and lethality in *egfp *TAL-injected embryos

Targeting nuclease(s)	Wild type	Abnormal	Dead	Total (N)
12.5 pg egfp5TAL	86 (93.5%)	6 (6.5%)	0 (0%)	92
25 pg egfp5TAL	14 (23.0%)	44 (72%)	3 (4.9)	61
12.5 pg egfp3TAL	89 (96.7%)	2 (2.2%)	1 (1.1%)	92
25 pg egfp3TAL	53 (61.6%)	28 (32.6%)	5 (58.0%)	86
12.5 pg egfp5TAL+egfp3TAL	202 (82.8%)	29 (11.9%)	13 (5.3%)	244
25 pg egfp5TAL+egfp3TAL	40 (33.1%)	62 (51.2%)	19 (15.7%)	121

Numbers were tabulated from at least two independent experiments.

PCR with primers flanking the TALEN sites (Figure 1a, blue and magenta triangles) shows an approximately 250 bp fragment in all injected embryos (*n *= 23), compared to a 854 bp wild-type e*gfp *fragment, indicating excision of intervening sequences in some cells of injected embryos (Figure 1d). Sequencing of PCR products from individual embryos showed large as well as small deletions, likely due to mosaicism of the injected nuclease RNA pairs, and non-homologous end joining events (Figure 1e; Figure S1B,C,E in Additional file 1). Comparison of sequences of single TALEN versus double TALEN pair injections shows lower deletion frequency with single TALEN pair injections, presumably because small deletions induced by single nuclease pairs are repaired more efficiently than the larger lesions induced by multiple TALEN pairs (Figure S1A-E,H in Additional file 1). Moreover, the majority of mutant alleles from double TALEN injections showed complete deletions (Figure S1B,C,E in Additional file 1). These results show that defined large deletions that disrupt target gene expression can be generated easily via the combinatorial action of multiple TALENs.

**Figure 2:**
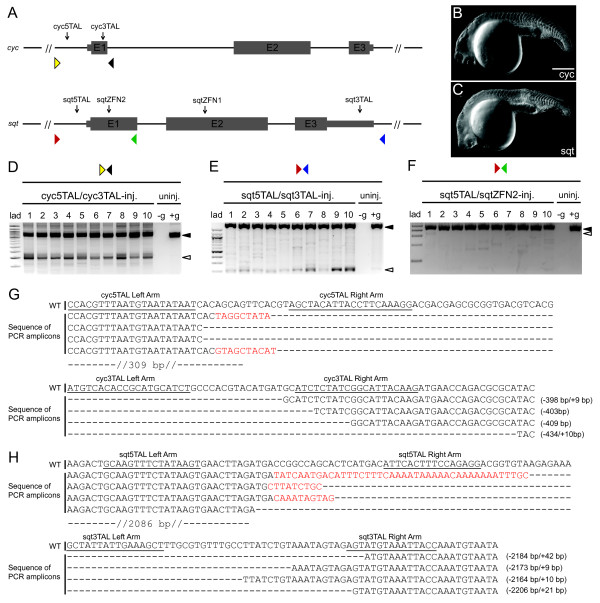
**Targeted deletions in *cyc *and *sqt *by multiple TALEN and ZFN pairs**. **(a) **Schematic representation of the *cyc *and *sqt *loci, with positions of the TALEN targeting sites and ZFN targeting sites indicated by black arrows. E1, E2 and E3 indicate *cyc *or *sqt *exons 1 to 3. Colored triangles in the both *cyc *and *sqt *panels indicate the position of primers used for genotyping. **(b) **Phenotype of *cyc *TALEN injected embryo at 24 hpf showing cyclopia. Scale bar, 100 μm. **(c) **Phenotype of representative *sqt *nuclease-injected embryo manifesting cyclopia and midline defects. **(d) **PCR with primers (yellow and black triangles in (a)) spanning the TALEN targeting sites (black arrows in (a)) shows the expected approximately 400 bp truncated *cyc *(white arrowhead), and 779 bp full-length *cyc *(black arrowhead) products in ten single embryos injected with *cyc *TALEN pairs, whereas the full-length product is observed in the un-injected control embryo. All embryos show faint intermediate sized products. No template control is indicated by -g. **(e) **PCR with primers (red and blue triangles in (a)) spanning the *sqt *locus show a 2.4 kb product (black arrowhead) for the intact *sqt *locus, whereas individual embryos with TALEN deletions show an approximately 220 bp complete locus deletion product (white arrowhead) and several other intermediate sized products. **(f) **PCR with primers spanning the *sqt *TSS site (red and green triangles in (a)) show a 478 bp full-length wild-type product (black arrowhead), and only one embryo (number 1) shows the expected approximately 300 bp deletion product (white arrowhead). **(g) **Alignment of wild-type (WT) *cyc *sequences with mutated PCR amplicons shows various deletions of approximately 400 bp between the targeting sites, accompanied by small insertions (red). **(h) **Alignment of wild type s*qt *sequences with mutated PCR amplicons shows various deletions of approximately 2.2 kb between the targeting sites, accompanied by small insertions (red).

Next, to determine if large deletions in endogenous loci and element-specific deletions can be generated effectively, we designed and synthesized one TALEN pair towards sequences approximately 230 bp upstream of the predicted transcriptional start site (TSS), and a second pair targeting sequences within *cyc *exon 1 (cyc5TAL, chr12: 49,427,780-49,427,835; cyc3TAL, chr12: 49,428,165-49,428,221), spanning a genomic region of approximately 380 bp that encompasses the TSS (Figure 2a). Similarly, to target *sqt*, we generated one TALEN pair specific to the 5' sequences upstream of the TSS, and a second pair targeting sequences in the 3' UTR of *sqt *(sqt5TAL, chr21: 19,838,706-19,838,767; sqt3TAL, chr21: 19,840,869-19,840,929; zebrafish genome assembly Zv9). The TALEN target sites span a chromosomal region of approximately 2.16 kb, encompassing the *sqt *gene. We also used sqt5TAL in conjunction with a ZFN pair targeting *sqt *exon 1 (sqtZFN2, Figure 2a), to delete a 98 bp genomic region surrounding the TSS (sqtZFN2, chr21: 19,838,905-19,838,934).

To determine the optimal dosage, we microinjected various concentrations of *sqt *TALEN and ZFN pairs, or *cyc *TALEN pairs into one-cell zebrafish embryos individually and in combinations, and assessed the cutting efficiency, phenotypes and survival at 24 hpf (Table 3; Figures S1A-D,F-G,I-J and S2 in Additional file 1). Cyclopia and midline defects, phenotypes that are visible in *cyc *and *sqt *mutant embryos [23,25], were found at frequencies ranging from 13 to 40% for *cyc *and 15 to 25% for *sqt*, indicating bi-allelic mutations in a proportion of injected F0 embryos (Figure 2b,c, Table 3; Figure S2 in Additional file 1). The efficacy of deletion mutations was assessed by PCR and sequencing from individual 1 dpf embryos (Figure 2d-f; Figure S1A-D,F-G,I-J in Additional file 1). Alignment to wild-type *sqt *genomic sequences showed that each TALEN and ZFN pair by itself generated small insertions and deletions of varying lengths (Figure S1D,I in Additional file 1), consistent with previous reports using single ZFN or TALEN pairs [9,10,26]. PCR performed on embryos injected with combinations of cyc5TAL and cyc3TAL, sqt5TAL and sqt3TAL, or sqt5TAL and sqtZFN2 showed both small and large fragments, including some of the size expected by targeted deletion of the intervening sequences (approximately 400 bp for *cyc*, approximately 220 bp for *sqt *whole locus deletion, approximately 300 bp for *sqt *TSS deletion), although the sqt5TAL+sqtZFN2 pair was not as efficient as the other double nuclease pair injections (Figure 2d-f; Figure S1B,C,F,G in Additional file 1). Sequencing of the PCR amplicons and alignment to wild-type *cyc *and *sqt *sequences shows that large deletions can be accompanied by insertions at both 5' and 3' targeting sites, indicative of non-homologous end joining events (Figure 2G,H; Figure S1B,C,F,G in Additional file 1). These results show that large defined deletions in endogenous loci can be generated efficiently by using multiple targeting nucleases, and result in mutant phenotypes. Furthermore, TALENs can be used simultaneously with ZFNs to generate chromosomal lesions (Figures S4 and S5 in Additional file 1).

**Table 3:** Frequency of cyclopia and mid-line defects in *cyc *and *sqt *nuclease-injected embryos

Targeting nuclease(s)	Wild type	Cyclopia and midline defects	Abnormal	Dead	Total (N)
6.25 pg cyc5TAL+cyc3TAL	176 (77.5%)	30 (13.2%)	8 (3.5%)	13 (5.8%)	227
12.5 pg cyc5TAL+cyc3TAL	134 (60.1%)	54 (24.2%)	17 (7.6%)	18 (8.1%)	223
25 pg cyc5TAL+cyc3TAL	11 (16.2%)	27 (39.7%)	27 (39.7%)	3 (4.4%)	68
25 pg sqtZFN2	62 (53.0%)	25 (21.4%)	18 (15.4%)	12 (10.2%)	117
50 pg sqtZFN2	32 (29.1%)	26 (23.6%)	37 (33.6%)	15 (13.6%)	110
25 pg sqt5TAL	22 (71.0%)	3 (9.7%)	0 (0.00%)	6 (19.3%)	31
50 pg sqt5TAL	43 (45.7%)	15 (16.0%)	18 (19.1%)	18 (19.1%)	94
25 pg sqt3TAL	18 (47.4%)	0 (0.00%)	5 (13.1%)	15 (39.5%)	38
50 pg sqt3TAL	29 (38.2%)	12 (15.8%)	15 (19.7%)	20 (26.3%)	76
25 pg sqt5TAL+sqtZFN2	22 (29.3%)	17 (22.7%)	19 (25.3%)	17 (22.7%)	75
25 pg sqt5TAL+sqt3TAL	30 (36.1%)	20 (24.1%)	15 (18.1%)	18 (21.7%)	83

Numbers were tabulated from at least two independent experiments.

To determine the germ-line transmission frequency of the deletion mutations, we raised *sqt *and *cyc *nuclease-injected embryos to adulthood, and screened their progeny by PCR with primers spanning the targeting sites (colored triangles in Figure 2a; Table S2 in Additional file 1). For *cyc*, we observed deletions in 4.5 to 23% F1 progeny of 10/36 F0 founders. Of these, 9/10 founders yielded embryos with complete TSS deletions, 1/10 showed a smaller deletion near the cyc3TAL target site, and the same founder also transmitted a second mutation comprising a 187 bp deletion near the cyc5TAL target site, together with a 174 bp inversion and a 14 bp insertion near the 3' cyc3TAL target site (Table 4; Figure S3 in Additional file 1). In 3/10 founders, we also observed multiple mutation events (Figure S3 in Additional file 1). We identified *sqt *whole-locus deletions in 3.3 to 9.5% F1 progeny of 6/56 F0 founders injected with the sqt5TAL and sqt3TAL pairs. The smaller 5' TSS deletions generated with the sqt5TAL and sqtZFN2 pairs were observed in 3.3 to 6.7% F1 embryos from 2/28 F0 fish (Table 3; Figure S4A,B in Additional file 1). However, of the two founders with the *sqt *TSS deletion, only one appears to have been due to targeting by both sqt5TAL and sqtZFN2 pairs, whereas the other is likely from activity of the sqt5TAL alone (Figures S4 and S5 in Additional file 1). These results suggest that *cyc *is targeted at higher efficiency than *sqt *by the nucleases. For *sqt*, the efficiency and the germ-line transmission frequency of the larger *sqt *whole locus deletions are not substantially different from that of the smaller *sqt *TSS deletions.

**Table 4:** Germ-line transmission frequency of *cyc *and *sqt *nuclease-induced lesions in zebrafish

Targeting nuclease(s)	Number of F_0 _screened	Number of mutant F_0_s
sqt5TAL + sqt3TAL	56	6 (whole locus deletions)
sqt5TAL + sqtZFN2	28	2 (TSS deletions)
sqtZFN1	92	1 (4 bp insertion)
cyc5TAL + cyc3TAL	36	10 (9 founders with TSS deletions, and 1 with a non-TSS 151 bp deletion and a deletion + inversion + insertion)

For each founder (F_0_), at least 30 F1 embryos for *sqt*, or 22 F1 embryos for *cyc *were analyzed.

**Figure 3:**
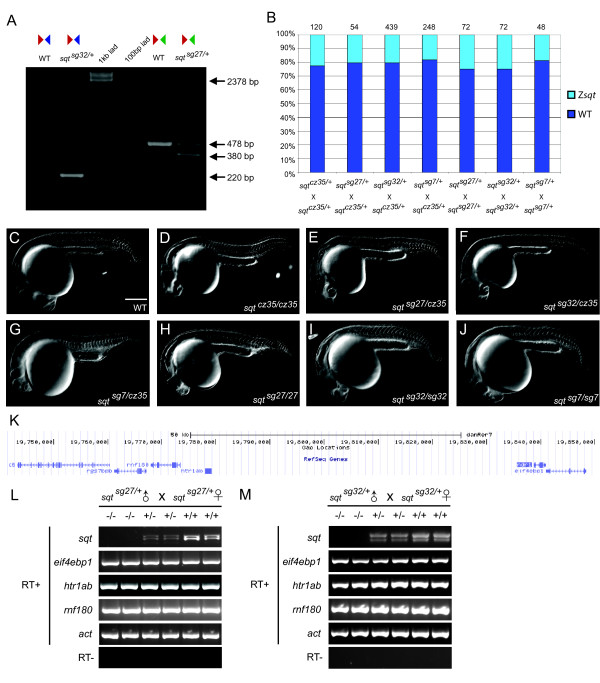
**Heritable deletions in the *sqt *locus that result in RNA-null alleles**. **(a) **PCR on single wild-type or *sqt *deletion mutant embryos (using primers indicated in Figure 2a) shows a 220 bp fragment in a s*qt^sg32 ^*locus-deletion embryo, and a 380 bp fragment in TSS deleted *sqt^sg27 ^*mutant embryo. Sometimes a larger approximately 500 bp fragment is observed in *sqt^sg27^*/+ heterozygous embryos, but the sequence is identical to the 478 bp product. **(b) **Percentage of embryos with *sqt *mutant phenotypes in *sqt ^cz35/+^*, *sqt ^sg27/+^*, *sqt ^sg32/+ ^*and *sqt ^sg7/+ ^*in-crosses and mating of *sqt ^cz35/+ ^*with *sqt ^sg27/+^*, *sqt ^sg32/+ ^*and *sqt ^sg7/+^*. The *cz35 *allele is an approximately 1.9 kb insertion in *sqt *exon 1; the *sg27 *allele is a 98 bp deletion of *sqt *TSS sequences; *sg32 *allele is a whole locus deletion of *sqt*; the *sg7 *ZFN allele harbors a GGCC insertion in *sqt *exon 2. **(c-j) **DIC images of 24 h wild-type (c), *sqt ^cz35/cz35 ^*(d), *sqt ^sg27/cz35 ^*(e), *sqt ^sg32/cz35 ^*(f), *sqt ^sg7/cz35 ^*(g), *sqt ^sg27/sg27 ^*(h), *sqt ^sg32/sg32 ^*(i), and *sqt ^sg7/sg7 ^*(j) embryos; scale bar in (c), 100 μm. **(k) **UCSC genome browser view of the *sqt *locus and neighboring genomic region. **(l,m) **RT-PCR with primers to detect expression of *sqt *RNA and transcripts of neighboring genes, *eif4ebp1*, *rnf180*, and *htr1ab*, shows lack of *sqt *RNA expression in *sqt ^sg27/sg27 ^*(l) and *sqt ^sg32/sg32 ^*(m) embryos whereas all neighboring gene transcripts are expressed at wild-type levels. Actin (*act*) expression was used as control. In contrast, both un-spliced and spliced *sqt *RNA is detected in wild-type and heterozygous embryos.

We then examined embryos obtained by mating fish heterozygous for the ZFN-induced *sqt^sg7^*, sqt5TAL*/*sqt3TAL-induced *sqt^sg32 ^*whole-locus or sqt5TAL*/*sqtZFN2-induced *sqt^sg27 ^*TSS deletion mutations with *sqt^cz35 ^*insertion mutant carriers [25], and found embryos that manifest cyclopia and deficiencies in midline structures such as the notochord (Figure 3a-j). Therefore, the *sqt *TALEN and ZFN-induced lesions do not complement the *sqt^cz35 ^*insertion mutant phenotypes.

We then ascertained if the *sqt *TSS and whole locus deletions actually abolish *sqt *RNA expression in mutant embryos. We also determined if adjacent genomic regions and elements were affected by the *sqt *deletions, by examining expression of neighboring genes (*eif4ebp1*, *htr1ab*, and *rnf180*; Figure 3k) at appropriate stages. RT-PCR analyses to detect expression of immediate flanking loci show that their transcription is unaffected in the *sqt^sg27 ^*TSS deletion mutant embryos (Figure 3l). By contrast, expression of *sqt *RNA is significantly reduced in embryos heterozygous for the *sqt^sg27 ^*TSS deletion mutation, and is not detected in homozygous *sqt^sg27 ^*embryos (Figure 3l). Similarly, *sqt *RNA expression is not detected in homozygous *sqt^sg32 ^*whole-locus deletion mutant embryos, whereas flanking gene expression is unaffected (Figure 3m). Thus, our *sqt *deletions do not affect neighboring transcriptional units and these deletions are bona fide *sqt *RNA-null alleles.

## Discussion

Our method demonstrates the ease of generating heritable whole locus deletions by the combinatorial action of multiple targeting nucleases. The ability to easily create targeted, heritable deletions in animal models such as zebrafish will greatly facilitate generation and analysis of humanized deletion mutations such as those observed in patients with hereditary neuropathies or polydactyly [27,28]. Therefore, we believe our strategy can be applied in a variety of organisms, including those (for example, the mouse) in which current methods for chromosomal engineering employ the labor-, time- and resource-intensive strategy of first generating targeted insertions by homologous recombination, and then editing via Cre/Lox or Flp/FRT systems. Recently, Zu *et al. *[29] described a method using TALENs to make precise modifications by homologous recombination in zebrafish. This is an immensely valuable method for directed genome engineering, and could potentially also be used to generate precise segmental deletions by engineering loxP sites at desired locations on chromosomes [29,30]. However, the germ-line transmission efficiency of targeted homologous recombination by this method is currently approximately 1.5%. In contrast, our method to generate deletions by direct targeting of chromosomal segments using multiple targeting nucleases is efficient, and does not require introduction of Cre recombinase or breeding for additional generations (Table 5). Therefore, combinations of targeted nucleases can be used to rapidly generate chromosomal deletions at predetermined locations.

**Table 5:** Mutation frequency of double nuclease pairs versus homology directed repair

	Clone size	Percentage of positive founders	Founders screened (n)	Source
*cyc Δ*TSS	4.5-22.5%	27.8%	36	This study
*sqt Δ*WL	3.3-9.5%	10.7%	56	This study
*sqt Δ*TSS	3.3-6.7%	7.1%	28	This study
*apoea -477bp*	2-11%	31.3%	16	Gupta *et al*. 2013 [13]
*apoea -4.2kb*	1-13%	29.4%	17	Gupta *et al*. 2013 [13]
*th *HDR	6.0-29.7%	1.5%	275	Zu *et al*. 2013 [29]
*ponzr1 *HDR	NA	~1.6%	186	Bedell *et al*. 2012 [30]
*crhr2 *HDR	NA	~13.8%	58	Bedell *et al*. 2012 [30]

Frequency of deletions in whole locus (*Δ*WL) and transcriptional start site (*Δ*TSS) for *cyc *and *sqt *compared to large deletions in *apoea*, and homology directed repair (HDR) at the *th*, *ponzr *and *crhr2 *loci are shown. NA, data not available.

Chromosomal deletions can be used for analyzing gene clusters and regulatory regions, and for determining the functions of non-coding as well as coding RNAs in the genome. In support of this possibility, our *sqt^sg27 ^*TSS deletion that is predicted to excise the TSS elements and *sqt^sg32 ^*whole-locus deletion indeed result in mutant embryos that are *sqt *RNA-null. Furthermore, zygotic *sqt^sg27 ^*and *sqt^sg32 ^*deletion mutant embryos manifest phenotypes that are consistent with the previously identified *sqt *mutations. Thus, this strategy can be used effectively to investigate the roles of all 'functional' RNAs in the genome.

The various targeting nucleases have different constraints pertaining to target sites. For instance, TALENs prefer a 5' T nucleotide, whereas CRISPR/Cas9 requires a GG dinucleotide for targeting. The spacer requirements for the various nucleases are also different, and targetable sites for the different nucleases likely occur at different frequencies in genomes [31,32]. Therefore, using the combinatorial action of various nucleases can facilitate generation of defined deletions at desired locations with higher efficacy. Moreover, some TALEN and ZFN sites (for instance, our *sqt *ZFN target sites) are just not targeted efficiently for reasons that are still unclear. Hence, the ability to use multiple targeting nucleases in various combinations offers additional flexibility and alternative approaches to engineer chromosomes than is possible with individual nuclease pairs. The efficiency and precision of the deletion events can be improved further by using nuclease variants such as the 'GoldyTALEN' system [30].

Our simple, facile and efficient strategy is largely PCR-based, and, therefore, can be used with modest resources to generate deletion mutants for investigating functional elements in the genome. Finally, this approach of generating large, defined heritable deletions by simultaneously targeting two discrete regions on a single chromosome can potentially also be deployed with RNA-guide mediated or other emerging DNA cleavage methods [32-34] to enhance the toolkit for heritable chromosomal engineering in a variety of organisms.

## Conclusions

Targeted and heritable chromosomal deletions can be rapidly generated in a whole organism by using the combinatorial action of targeting nucleases. Multiple nuclease pairs are apparently more effective than single nuclease pairs in generating targeted deletions. Whole-locus as well as TSS element-specific deletions were generated efficiently by this method, and stably transmitted through the germ-line. The deletion mutations result in transcript-null alleles that manifest embryonic mutant phenotypes, demonstrating functional consequences of the chromosomal lesions. This simple, facile and efficient strategy can be used with modest resources. Thus, our strategy can be used to generate disease models, and for analysis of gene clusters, regulatory regions and functional RNAs in the genomes of a variety of organisms.

## Materials and methods

### Generation of plasmids encoding TALENs and ZFNs

The *egfp*, *sqt *and *cyc *TALENs target sites were designed using an online tool [35]. To check for unique targeting sites, BLAST and UCSC BLAT search was performed with the zebrafish genome assembly (Zv9) using the target site sequences. The TAL effector repeats were constructed from four TAL effector single unit vectors (pA, pT, pG^NN ^and pC) using the 'unit assembly' method [9]. Plasmids encoding sqtZFN1 and sqtZFN2 nuclease pairs were obtained from ToolGen, Inc. (Seoul, South Korea). The TALEN and ZFN target sites for *egfp*, *cyc *and *sqt *are shown in Figures 1a and 2a and sequences are listed in Table S1 in Additional file 1.

### TALEN and ZFN capped mRNA synthesis

Using the Ambion® SP6 mMESSAGE mMACHINE kit (Life Technologies, Carlsbad, California, United States of America), capped TALEN mRNAs were transcribed *in vitro *from 1.0 µg of the respective *Not*I linearized TALEN expression vectors. To synthesize capped ZFN mRNAs, *sqt*-specific ZFN plasmids were linearized with *Xho*I and transcribed using T7 RNA polymerase (Promega, Fitchburg, Wisconsin, United States of America). RNA was purified by phenol-chloroform precipitation and dissolved in RNase-free water.

### Microinjection of capped TALEN and ZFN mRNA into zebrafish embryos

All experiments using animals were performed in accordance with institutional animal care and use guidelines. For *sqt *and *cyc *experiments, embryos from wild type (AB) fish were used for injections. For *egfp *TALEN experiments, embryos from *Tg (Ds DELGT4) ^sg310 ^*homozygous males mated with wild-type AB females were used. *Tg (Ds DELGT4) ^sg310 ^*transgenic fish harbor a Ds transposon-mediated enhancer trap insertion on chromosome 21. Various dosages and combinations of nuclease RNAs were tested to determine the toxicity, and the maximum dose that yielded less than 50% lethality was used (Table 2). For testing single TALEN or ZFN pairs, either 12.5, 25 or 50 pg of each mRNA was injected into one-cell stage zebrafish embryos. Higher lethality rates and abnormal embryos were observed with the *cyc *TALEN pairs and, therefore, cyc5TAL and cyc3TAL mRNAs were introduced at 6.25, 12.5 or 25 pg doses per embryo. For double TALEN pair or TALEN+ZFN experiments, a cocktail of either 12.5 or 25 pg of each mRNA was injected into one-cell stage zebrafish embryos. Injected embryos were examined at prim-5 stage under a Leica MZ12.5 stereomicroscope. PCR products from individual embryos injected with each single nuclease pair were tested by the T7E1 assay and sequencing to assess the efficacy of each nuclease pair. Ten embryos that were morphologically normal were selected and processed for PCR and sequencing. The remaining embryos were raised to adulthood to determine the germ-line transmission rates.

### PCR and sequence analyses

To detect deletions in founder (F_0_) embryos, at least 10 TALEN- and ZFN-injected embryos were individually lysed at 24 hpf in 20.0 µl of DNA extraction buffer (10 mM Tris pH 8.2, 10 mM EDTA, 200 mM NaCl, 0.5 % SDS, 100 µg/ml proteinase K) for 5 h at 55°C, followed by heat inactivation of proteinase K at 65°C for 10 minutes. Genomic DNA was diluted five-fold using 1× TE Buffer (pH 8.0), and 2 µl aliquots were used in 20 µl PCR reactions. For single nuclease pair experiments, fragments containing 100 to 150 bp upstream and downstream of the expected target sites were amplified with Go Taq polymerase (Promega). For double TALEN or TALEN+ZFN experiments, primers annealing to regions 100 to 150 bp upstream of 5' TALEN and downstream of the 3' TALEN or ZFN target sites were used in PCR from genomic DNA template using Phusion® High-Fidelity polymerase (New England Biolabs, Ipswich, Massachusetts, United States of America) following the manufacturer's instructions (the primers used are listed in Table S2 in Additional file 1**)**. Five microliter aliquots of products from ten single embryo PCRs were pooled, gel purified to remove primer dimers and cloned into either Promega pGEM®-T easy TA cloning vector or Fermentas pJET1.2 blunt end cloning vector, and transformed using XL1-blue heat-shock competent bacterial cells. At least 48 bacterial colonies were picked for screening by PCR. PCR products were diluted three-fold, and 1 μl was used directly for sequencing using the same primer pairs. Sequences were analyzed by comparison to the Zv9 Zebrafish Genome Assembly.

### T7E1 assay to detect indels induced by single nuclease pairs

Five microliter aliquots of single embryo PCR products were diluted to 20 μl in 1× NEB Buffer 2, denatured at 95°C for 5 minutes, slowly cooled to room temperature to allow annealing and formation of hetero-duplexes. The individual preps were then treated with 5 units of T7E1 (New England Biolabs) for 30 minutes at 37°C. Digested products were separated on a 3.5% agarose/1×TBE gel and band intensity analyzed using ImageJ (NIH) to calculate mutation frequencies [36].

### Genotyping of F1s

To assess the germ-line transmission rates, injected F_0 _fish were raised to adulthood, and mated either with siblings or wild-type fish to obtain F_1 _progeny. For genotyping *sqt *nuclease- or *cyc *TAL-injected embryos, PCR was performed using primers listed in Table S2 in Additional file 1, and Taq polymerase (Promega). PCR amplicons were electrophoresed on a 2% agarose gel. To screen for germ-line transmission events at the endogenous *sqt *locus, we analyzed progeny from pairwise mating of founders. Single embryos from six founder fish (three pairs) were screened per 96-well plate. At least 30 embryos (24 hpf) from each mating were collected, lysed and analyzed by PCR using the same primer pairs as used for the transient assays. This number allowed efficient detection of germ-line transmission events (whose frequency ranged from 3 to 10%), and recovery of the mutation. Bands of aberrant sizes were either sequenced directly or after cloning into the pGEM®-T easy vector system. F_1 _progeny of positive F_0_s were raised to adulthood, and heterozygous carriers for the deletions were identified by fin-clipping and routine genotyping PCR analysis, using primers listed in Table S2 in Additional file 1. The *sqt^sg7 ^*ZFN1-induced allele harbors a 4 bp insertion in exon2 (chr21: 19839892-19839896; Figure S5 in Additional file 1). The *sg7 *mutation is predicted to result in a frame-shift after amino acid 143 in Sqt protein and premature termination after amino acid residue 146. Homozygous *sqt^sg7 ^*embryos express *sqt *RNA [37]. The *sqt^sg27 ^*mutants harbor an indel (chr21: 19838727-1983870; Figure S5 in Additional file 1) and lack the transcriptional start sequences, and the lesion in *sqt^sg32 ^*is a whole locus deletion of 2.1 kb on chromosome 21 (Figure S5 in Additional file 1). For analyzing germ-line transmission rates of *cyc *deletions, we collected progeny from pairwise mating of founders in pools of five embryos since the somatic mutation frequency for the *cyc *TALENs was higher than that for *sqt*. At least ten pools from each successful mating were collected and used in PCRs to ensure that founders with mutant clone sizes larger than 2% were identified. Subsequently, founders that yielded mutations were mated with wild-type (AB) fish. At least 22 single embryos from each mating were collected for PCR and sequencing to confirm and determine the germ-line transmission rate. (For a list of primers, see Table S2 in Additional file 1.)

### Semi-quantitative RT-PCR

Using TRIzol reagent (Invitrogen, Carlsbad, California, United States of America), both genomic DNA and total RNA were extracted from single 30% epiboly stage and 2 dpf (for *htr1ab *expression) embryos obtained from heterozygous *sqt^sg27/+ ^*and *sqt^sg32/+ ^*crosses. For genotyping, 50 ng of genomic DNA was used as template in 20 µl PCR reactions. For first-strand cDNA synthesis, 250 ng of total RNA was used in a pdN6-primed reaction using SuperScript® II Reverse Transcriptase (Life Technologies). First-strand cDNA (1 µl) was used in 20 µl PCR reactions to detect expression of *sqt*, *ring finger protein *(*rnf180*), *5-hydroxytryptamine (serotonin) receptor 1A b *(*htr1ab*), *eukaryotic translation initiation factor 4E binding protein 1 *(*eif4ebp1*) and control *actin *(*act*), using the primers listed in Table S3 in Additional file 1.

### Microscopy

Embryos were manually de-chorionated using fine forceps and mounted in 2.5% methylcellulose on a depression slide. DIC images were captured using a monochrome CoolSNAP HQ camera (Photometrics, Tucson, Arizona, United States of America) fitted on a Zeiss Axioplan2 upright microscope. The *egfp *TALEN injected and un-injected *Tg (Ds DELGT4) ^sg310 ^*embryos were manually de-chorionated and mounted in 1.5% low melting agarose (Bio-Rad, Hercules, California, United States of America) on tissue culture dishes with cover-slip bottoms (World Precision Instruments, Inc. FluoroDish FD3510-100, Sarasota, Florida, United States of America). Images were captured using a Leica SP5 inverted confocal system.

## Abbreviations

EGFP: enhanced green fluorescent protein; hpf: hours post-fertilization; PCR: polymerase chain reaction; T7E1: T7 endonuclease I; TALEN: transcription activator-like effector nuclease; TSS: transcriptional start site; UTR: untranslated region; ZFN: zinc finger nuclease.

## Competing interests

The authors declare that they have no competing interests.

## Authors' contributions

Experimental design: SL, YW, KS. Performed experiments: XY, YH, YW, SL, KS. Wrote the manuscript: SL, YW, MF, KS. All authors read and approved the final manuscript.

## Supplementary Material

Additional file 1 **Figures S1 to S5 and Tables S1 to S3**. Figure S1: **(A) **graph showing percentage cutting efficiency of single TALEN pairs targeting *egfp*, *sqt *and *cyc*, in comparison to double TALEN pairs, as determined by sequencing (percentage 5'-3' complete deletions amongst all sequenced alleles for double TAL pair injections are shown as purple bars). **(B) **Graph showing frequency of different mutant alleles in double TALEN pair-injected embryos for *egfp*, *sqt *and *cyc*. Deletions in the 5' site alone, 3' site alone, complete 5'-3' deletions, and incomplete 5'-3'deletions (which are larger than individual 5' alone or 3' alone deletions but smaller than complete 5'-3' deletions) were observed. **(C) **Table showing frequency of mutations induced by double nuclease pair injections as a percentage of total number of alleles. **(D) **Table showing frequency of mutations induced by single nuclease pair injections. **(E-J) **Alignment of *egfp*, *sqt *and *cyc *sequences from single or double nuclease pair injected embryos showing 5' only, 3' only, and incomplete or complete 5'-3' deletions. For double-nuclease pair injections, the target site of the 5' pairs is highlighted in yellow and the 3' pairs in green. Insertions are highlighted in blue and deletions are indicated with red dashed lines. Numbers in the middle of the alignment indicate the number of intervening gaps and bases. Single nucleotide substitutions are highlighted in magenta. Nature and extent of mutations, and frequency of alleles observed >1 (× n) are shown to the right of the alignments. Figure S2: representative phenotypes observed at 24 h in embryos injected with *sqt *or *cyc *TALENs. At high doses, the proportion of abnormal embryos increases. Figure S3: **(A) **Alignment of *cyc *sequences showing TSS deletions in embryos from F_0 _founders, compared to wild-type *cyc*. Insertions are indicated in red, and gaps are shown by dashed lines. Letter suffixes (for example, 1A and 1B) represent different alleles from the same founder. Eight founders injected with *cyc *TALENs transmitted complete deletion of the intervening sequences, some of which also show insertion events. Founder F0-4, shows a larger deletion that extends beyond the 3' end of the cyc3TAL target site. Genomic coordinates on chromosome 12 for wild-type *cyc *are indicated for the regions shown. **(B) **Alignment of *cyc *sequence of F0-10 to wild-type *cyc *shows a deletion (dashed lines), accompanied by an inversion (yellow highlight) and insertion (red font). Figure S4: **(A) **Alignment of *sqt *sequences showing the whole-locus and TSS deletions in embryos from F_0 _founders, compared to wild-type *sqt*. Insertions are highlighted in red font, and gaps are indicated with dashed lines. All six founders for sqt5TAL/sqt3TAL showed embryos with the 2.1 kb whole locus deletion. **(B) **For sqt5TAL/sqtZFN2, founder 1 (F0-1) showed embryos with the intervening sequences excised, whereas founder 2 (F0-2) sequences indicate that only sqt5TAL was active. Genomic coordinates on chromosome 21 for wild-type *sqt *are indicated for the regions shown. TALEN and ZFN target sites are indicated. Figure S5: alignment of *sqt *sequences showing the *sqt^sg27 ^*TSS deletion **(A)**, *sqt ^sg32 ^*whole-locus deletion **(B)**, and *sqt^sg7 ^*ZFN **(C) **mutations in comparison to wild-type sqt. Insertions are highlighted in red font, and gaps are indicated with dashed lines. TALEN and ZFN target sites are indicated. Table S1: list of target sites for TALENs and ZFNs. Table S2: list of primers for genotyping, sequencing, and T7E1 assays. Table S3: list of primers for detecting expression of *sqt*, *rnf180*, *htr1ab*, *eif4ebp1 *and *act*. Click here for file
