# Supplementary material for: A simple strategy for heritable chromosomal deletions in zebrafish via the combinatorial action of targeting nucleases
Source: Genome Biol. 2013 Jul 1;14(7):R69. doi: 10.1186/gb-2013-14-7-r69 (PMC4054832; doi:10.1186/gb-2013-14-7-r69)
Supplement: Additional file 1 — Figures S1 to S5 and Tables S1 to S3. Figure S1: (A) graph showing percentage cutting efficiency of single TALEN pairs targeting egfp, sqt and cyc, in comparison to double TALEN pairs, as determined by sequencing (percentage 5'-3' complete deletions amongst all sequenced alleles for double TAL pair injections are shown as purple bars). (B) Graph showing frequency of different mutant alleles in double TALEN pair-injected embryos for egfp, sqt and cyc. Deletions in the 5' site alone, 3' site alone, complete 5'-3' deletions, and incomplete 5'-3'deletions (which are larger than individual 5' alone or 3' alone deletions but smaller than complete 5'-3' deletions) were observed. (C) Table showing frequency of mutations induced by double nuclease pair injections as a percentage of total number of alleles. (D) Table showing frequency of mutations induced by single nuclease pair injections. (E-J) Alignment of egfp, sqt and cyc sequences from single or double nuclease pair injected embryos showing 5' only, 3' only, and incomplete or complete 5'-3' deletions. For double-nuclease pair injections, the target site of the 5' pairs is highlighted in yellow and the 3' pairs in green. Insertions are highlighted in blue and deletions are indicated with red dashed lines. Numbers in the middle of the alignment indicate the number of intervening gaps and bases. Single nucleotide substitutions are highlighted in magenta. Nature and extent of mutations, and frequency of alleles observed >1 (× n) are shown to the right of the alignments. Figure S2: representative phenotypes observed at 24 h in embryos injected with sqt or cyc TALENs. At high doses, the proportion of abnormal embryos increases. Figure S3: (A) Alignment of cyc sequences showing TSS deletions in embryos from F0 founders, compared to wild-type cyc. Insertions are indicated in red, and gaps are shown by dashed lines. Letter suffixes (for example, 1A and 1B) represent different alleles from the same founder. Eight founders injecte [file gb-2013-14-7-r69-S1.PDF]

This document contains Figure S1-S5 and Table S1-S3

A simple strategy for heritable chromosomal deletions in zebrafish via the combinatorial action of targeting nucleases

Shimin Lim[1#], Yin Wang[2,3#], Xueyao Yu[4], Yian Huang[5], Mark S. Featherstone[1], and Karuna Sampath[1,2,3].

Figure S1.

A. Graph showing % cutting efficiency of single TALEN pairs targeting *egfp*, *sqt* and *cyc*, in comparison to double TALEN pairs, as determined by sequencing (% 5'-3' complete deletions amongst all sequenced alleles for double TAL pair injections are shown as purple bars).

B. Graph showing frequency of different mutant alleles in double TALEN pair-injected embryos for *egfp*, *sqt* and *cyc*. Deletions in the 5'- site alone, 3'-site alone, complete 5'-3' deletions, and incomplete 5'-3'deletions (which are larger than individual 5' alone or 3' alone deletions but smaller than complete 5'-3' deletions) were observed.

C. Table showing frequency of mutations induced by double nuclease pair injections as a % of total number of alleles.

D. Table showing frequency of mutations induced by single nuclease pair injections.

E-J. Alignment of *egfp*, *sqt* and *cyc* sequences from single or double nuclease pair injected embryos showing 5'- only, 3'-only, and incomplete or complete 5'-3' deletions. For double-nuclease pair injections, the target site of the 5' pairs is highlighted in yellow and the 3' pairs in green. Insertions are highlighted in blue and deletions are indicated with red dashed lines. Numbers in the middle of the alignment indicate the number of intervening gaps and bases. Single nucleotide substitutions are highlighted in magenta. Nature and extent of mutations, and frequency of alleles observed >1 (x n) are shown to the right of the alignments.

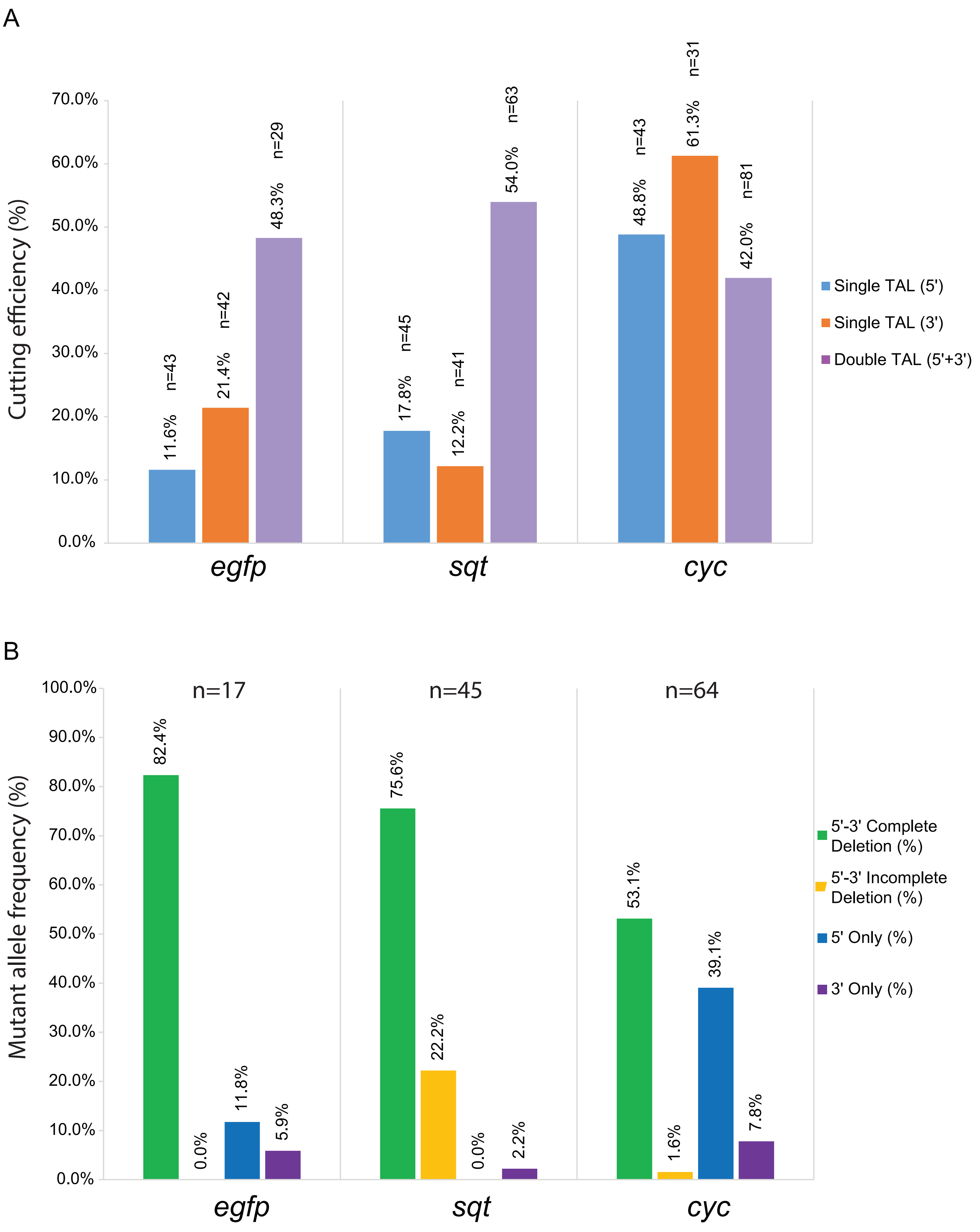

A
Cutting efficiency (%)
70.0%
60.0%
50.0%
40.0%
30.0%
20.0%
10.0%
0.0%
11.6% n=43
21.4% n=42
48.3% n=29
17.8% n=45
12.2% n=41
54.0% n=63
48.8% n=43
61.3% n=31
42.0% n=81
egfp
sqt
cyc
Single TAL (5')
Single TAL (3')
Double TAL (5'+3')
B
Mutant allele frequency (%)
100.0%
90.0%
80.0%
70.0%
60.0%
50.0%
40.0%
30.0%
20.0%
10.0%
0.0%
n=17
n=45
n=64
82.4%
0.0%
11.8%
5.9%
75.6%
22.2%
0.0%
2.2%
53.1%
1.6%
39.1%
7.8%
egfp
sqt
cyc
5'-3' Complete Deletion (%)
5'-3' Incomplete Deletion (%)
5' Only (%)
3' Only (%)

Figure S1.

C Mutations induced by double nuclease injections

Nuclease pair combinations	Dosage (pg)	Total Alleles (n)	WT Alleles (%)	Mutant Alleles				
				Total (%)	5'-3' Complete (%)	5'-3' Incomplete (%)	5' Only (%)	3' Only (%)
egfp5TAL+3TAL	12.5	29	41.4%	58.6%	48.3%	0.0%	6.9%	3.4%
sqt5TAL+3TAL	25	63	28.6%	71.4%	54.0%	15.9%	0.0%	1.6%
cyc5TAL+3TAL	12.5	81	19.8%	79.0%	42.0%	1.2%	30.9%	6.2%
sqt5TAL+ZFN2	25	45	100.0%	0.0%				

D Mutations induced by single nuclease injections

Nuclease pairs	Dosage	Total Alleles Sequenced	WT Alleles	Mutant Alleles	Mutation Frequency
egfp5TAL	12.5pg	43	38	5	11.6%
egfp3TAL	12.5pg	42	33	9	21.4%
sqt5TAL	25pg	45	37	8	17.8%
sqt3TAL	25pg	41	36	5	12.2%
sqtZFN2	25pg	45	40	5	11.1%
cyc5TAL	12.5pg	43	22	21	48.8%
cyc3TAL	12.5pg	31	12	19	61.3%

Figure S1.

E

egfp5TAL(12.5pg) + egfp3TAL(12.5pg)   No. of alleles: wt = 12   5’-3’ Complete = 14   5’-3’ Incomplete = 0   5’ short indel = 2   3’ short indel = 1

```
GTGAGCAAGGGCGAGGAGCTGTTCACCGGGGTGGTGCCCATCCTGGTCGAGCTGGACGGCGACGTAAACG//+560bp//CAACCACTACCTGAGCACCCAGTCCGCCCTGAGCAAAGACCCCAACGAGAAGCGCGATCACATGGTCCTGCTGGAGT      wt              x12

GTGAGCAAGGGCGAGGAGCTGTTCACCGGGGTGGTGCCCATCCTGGGTGGTACC----------------//-560bp//-----------------------------------------------------------CATGGTCCTGCTGGAGT      -596(-604,+8)
GTGAGCAAGGGCGAGGAGCTGTTCACCGGCCCCT------------------------------------//-560bp//------------------------------------------------GAAGCGCGATCACATGGTCCTGCTGGAGT      -604(-609,+5)
GTGAGCAAAGGCGAGGAGCTGTTCACC-------------------------------------------//-560bp//------------------------------------------CAACGAGAAGCGCGATCACATGGTCCTGCTGGAGT      -605
GTGAGCAAGGGCGAGGAGCTGTTCACCGGGTGG-------------------------------------//-560bp//----------------------------------------------------CGCGATCACATGGTCCTGCTGGAGT      -608(-612,+4)
GTGAGCAAGGGCGAGGAGCTGTTC----------------------------------------------//-560bp//-------------------------------------------------------GATCACATGGTCCTGCTGGAGT      -621
GTGAGCAAGGGCGAGGAGCTGTTCAC--------------------------------------------//-560bp//-------------------------------------------------------------ATGGTCCTGCTGGAGT      -625            x2
GTGAGCAAGGGCGAGGAGCTGTTCACCGGGGT--------------------------------------//-560bp//----------------------------------------------------------CACATGGTCCTGCTGGAGT      -616
GTGAGCAAGGGCGAG-------------------------------------------------------//-560bp//-------------------------------------------------AAGCGCGATCACATGGTCCTGCTGGAGT      -624
GTGAGCAAGGGCGAGGAGCT--------------------------------------------------//-560bp//------------------------------------------------------CGATCACATGGTCCTGCTGGAGT      -624
GTGAGCAAGGGCGAGGAGC---------------------------------------------------//-560bp//-------------------------------------------------------GATCACATGGTCCTGCTGGAGT      -626
GTGAGCAAGG------------------------------------------------------------//-560bp//-----------------------------------------------------------------TCCTGCTGGAGT      -645
GTGAGCAAGGGCGAGG------------------------------------------------------//-560bp//-------------------------------------------------------------//-16bp//--GGATT      -666
GTGAGCAAGGGCGAGGAGCTG-------------------------------------------------//-560bp//-------------------------------------------------------------//-63bp//--CGACT      -708

GAGCAAGGGCGAGGAGCTGTTCACCGGGGTGTGGTGCCCATCCTGGTCGAGCTGGACGGCGACGTAAACG//+560bp//CAACCACTACCTGAGCACCCAGTCCGCCCTGAGCAAAGACCCCAACGAGAAGCGCGATCACATGGTCCTGCTGGAGT      [5’]+2          x2

GTGAGCAAGGGCGAGGAGCTGTTCACCGGGGTGGTGCCCATCCTGGTCGAGCTGGACGGCGACGTAAACG//+560bp//CAACCACTACCTGAGCACCCAGTCCGCCCTGAGCAAAGACCCCAACG--GAGCGCGATCACATGGTCCTGCTGGAGT      [3’]-2(-3,+1)
```

Figure S1.

F

sqt5TAL(25pg) + sqt3TAL(25pg)   No. of alleles: wt = 18   5’-3’ Complete = 34   5’-3’ Incomplete = 10   5’ short indel = 0   3’ short indel = 1

```
CTGCAAGTTTCTATAAGTGAACTTAGATGACCGGCCAGCACTCATGACATTCACTTTCCAGAGGACGGT//    +2090bp   //AACTTGATGCTATTATTGAAAGCTTTGCGTGTTTGCCTTATCTGTAAATAGTAGAGTATGTAAATTACCAAA   wt               x18

CTGCAAGTTTCTATAAGTGAA------------------------------------------------//-2010bp, +79bp//AACTTGATGCTATTATTGAAAGCTTTGCGTGTTTGCCTTATCTGTAAATAGTAGAGTATGTAAATTACCAAA   -2058
CTGCAAGTTTCTATAAGTGAACTTAGATGTAATTTGAACTTAGAAACTTGCGTGTTTGCAT--------//    -2088bp   //----------------------------------------------AATAGTAGAGTATGTAAATTACCAAA   -2142(-2174,+32)
CTGCAAGTTTCTATAAGTGAACTTAGATGACTTAGATGAC-----------------------------//    -2088bp   //-------------------------------TTTGCCTTATCTGTAAATAGTAGAGTATGTAAATTACCAAA   -2147(-2156,+9)
CTGCAAGTTTCTATAAGTGAACTTAGATGACCGG-----------------------------------//    -2088bp   //----------------------------------GCCTTATCTGTAAATAGTAGAGTATGTAAATTACCAAA   -2157
CTGCAAGTTTCTATAAGTGAACTTAGATGACACTTAGA-------------------------------//    -2088bp   //--------------------------------------TATCTGTAAATAGTAGAGTATGTAAATTACCAAA   -2157(-2164,+7)
CTGCAAGTTTCTATAAGTGAACTTAGATGACCGGCC---------------------------------//    -2088bp   //-------------------------------------TTATCTGTAAATAGTAGAGTATGTAAATTACCAAA   -2158            x2
CTGCAAGTTTCTATAAGTGAACTTAGATGATTAGATTATCA----------------------------//    -2088bp   //-------------------------------------------GTAAATAGTAGAGTATGTAAATTACCAAA   -2159(-2170,+11)
CTGCAAGTTTCTATAAGTGAACTTAGAT-----------------------------------------//    -2088bp   //---------------------------------TGCCTTATCTGTAAATAGTAGAGTATGTAAATTACCAAA   -2162
CTGCAAGTTTCTATAAGTGAACTTAGATGACC-------------------------------------//    -2088bp   //-------------------------------------TTATCTGTAAATAGTAGAGTATGTAAATTACCAAA   -2162            x4
CTGCAAGTTTCTATAAGTGAACTTAGATGAC--------------------------------------//    -2088bp   //-------------------------------------TTATCTGTAAATAGTAGAGTATGTAAATTACCAAA   -2163
CTGCAAGTTTCTATAAGTGAACTTA--------------------------------------------//    -2088bp   //----------------------------------GCCTTATCTGTAAATAGTAGAGTATGTAAATTACCAAA   -2166
CTGCAAGTTTCTATAAGTGAACTTAGATGACCGGC----------------------------------//    -2088bp   //----------------------------------------------AATAGTAGAGTATGTAAATTACCAAA   -2168
CTGCAAGTTTCTATAAGTGAACTTAGATGACATATTAG-------------------------------//    -2088bp   //-------------------------------------------------AGTAGAGTATGTAAATTACCAAA   -2168(-2175,+7)
CTGCAAGTTTCTATAAGTGAACTTAGATGA---------------------------------------//    -2088bp   //--------------------------------------------TAAATAGTAGAGTATGTAAATTACCAAA   -2171            x2
CTGCAAGTTTCTATAAGTGAACTTA--------------------------------------------//    -2088bp   //----------------------------------------TCTGTAAATAGTAGAGTATGTAAATTACCAAA   -2172            x3
CTGCAAGTTTCTATAAGTGAACTTAGATG----------------------------------------//    -2088bp   //--------------------------------------------TAAATAGTAGAGTATGTAAATTACCAAA   -2172
CTGCAAGTTTCTATAAGTGAACTTAGATGTAAATAGTA-------------------------------//    -2088bp   //------------------------------------------------------AGTATGTAAATTACCAAA   -2173(-2182,+9)
CTGCAAGTTTCTATAAGTGAACTTAGA------------------------------------------//    -2088bp   //-----------------------------------------------ATAGTAGAGTATGTAAATTACCAAA   -2177
CTGCAAGTTTCTATAAGTGAACTTAGATGA---------------------------------------//    -2088bp   //-------------------------------------------------------GTATGTAAATTACCAAA   -2182
CTGCAAGTTTCTATAAGTGAACTTAGATGA---------------------------------------//    -2088bp   //------------------------------------------------------------TAAATTACCAAA   -2187
CTGCAAGTTTCTATAAGTGAACTTAGATGATTAGA----------------------------------//    -2088bp   //-------------------------------------TTATCTGTAAATAGTAGAGTATGTAAATTACCAAA   -2159(-2164,+5)
CTGCAAGTTTCTATAAGTGAACTTAGATGACC-------------------------------------//    -2088bp   //------------------------------------CTTATCTGTAAATAGTAGAGTATGTAAATTACCAAA   -2161
CTGCAAGTTTCTATAAGTGAACTTAGATGACC-------------------------------------//    -2088bp   //-------------------------------------TTATCTGTAAATAGTAGAGTATGTAAATTACCAAA   -2162            x2
CTGCAAGTTTCTATAAGTGAACTTAGAT-----------------------------------------//    -2088bp   //---------------------------------TGCCTTATCTGTAAATAGTAGAGTATGTAAATTACCAAA   -2162
CTGCAAGTTTCTATAAGTGAACTTAGAACT---------------------------------------//    -2088bp   //-------------------------------------TTATCTGTAAATAGTAGAGTATGTAAATTACCAAA   -2167(-2170,+3)
CTGCAAGTTTCTATAAGTGAACTTAGATGACCGG-----------------------------------//    -2088bp   //-----------------------------------------------ATAGTAGAGTATGTAAATTACCAAA   -2170

CTGCAAGTTTCTATAAGTGAACTTAGATGACCGGAGGAGGA----------------------------//-1095bp, +994bp//AACTTGATGCTATTATTGAAAGCTTTGCGTGTTTGCCTTATCTGTAAATAGTAGAGTATGTAAATTACCAAA   -1123(-1130,+7)
CTGCAAGTTTCTATAAGTGAACTTAGATGACCGGCCAGCACT---------------------------//-2105bp, +444bp//AACTTGATGCTATTATTGAAAGCTTTGCGTGTTTGCCTTATCTGTAAATAGTAGAGTATGTAAATTACCAAA   -1672
CTGCAAGTTTCTATAAGTGAACTTA--------------------------------------------//-1638bp, +451bp//AACTTGATGCTATTATTGAAAGCTTTGCGTGTTTGCCTTATCTGTAAATAGTAGAGTATGTAAATTACCAAA   -1682
CTGCAAGTTTCTATAAGTGAACTCTAATGTTTGTTG---------------------------------//-1783bp, +306bp//AACTTGATGCTATTATTGAAAGCTTTGCGTGTTTGCCTTATCTGTAAATAGTAGAGTATGTAAATTACCAAA   -1816(-1829,+13)
CTGCAAGTTTCTATAAGTGAACTTAGATGACCGGCCAGCACTCATGACATTCACTTTCCAGAGGACGGT//+230bp, -1859bp//----------------------------------------------AATAGTAGAGTATGTAAATTACCAAA   -1904
CTGCAAGTTTCTATAAGTGAACTTAGATG----------------------------------------//-1924bp, +165bp//AACTTGATGCTATTATTGAAAGCTTTGCGTGTTTGCCTTATCTGTAAATAGTAGAGTATGTAAATTACCAAA   -1964
CTGCAAGTTTCTATAAGTGAACTTAGATGACCGGCCAGCACTCATGACATTCACTTTCCAGAGGACGGT//+170bp, -1919bp//----------------------------------------------AATAGTAGAGTATGTAAATTACCAAA   -1964            x2
CTGCAAGTTTCTATAAGTGAACTTAGATGACCGGCCAG-------------------------------//-1934bp, +155bp//AACTTGATGCTATTATTGAAAGCTTTGCGTGTTTGCCTTATCTGTAAATAGTAGAGTATGTAAATTACCAAA   -1965            x2

CTGCAAGTTTCTATAAGTGAACTTAGATGACCGGCCAGCACTCATGACATTCACTTTCCAGAGGACGGT//    +2088bp   //AACTTGATGCTATTATTGAAAGCTTTGCGTGTTTGCCT--------AATAGTAGAGTATGTAAATTACCAAA   [3']-8
```

Figure S1.

G

cyc5TAL(12.5pg) + cyc3TAL(12.5pg)   No. of alleles: wt = 16   5’-3’ Complete = 34   5’-3’ Incomplete = 1   5’ short indel = 25   3’ short indel = 5

```
AGTGCAGCAAAGTATCCACGTTTAATGTAATATAATCACAGCAGTTCACGTAGCTACATTACCTTCAAAGGACGAGCGCGGTGACGTCACGCGCG//   +300bp   //GGAATATGTCACACCGCATGCATCTGCCCACGTACATGATGCATCTCTATCGGCATTACAAGATG     wt               x16

AGTGCAGCAAAGTATCCACGTTTAATGTAATATAATCACAATTATAC------------------------------------------------//-235bp, +65bp//GGAATATGTCACACCGCATGCATCTGCCCACGTACATGATGCATCTCTATCGGCATTACAAGATG     -283(-290,+7)
AGTGCAGCAAAGTATCCACGTTTAATGTAATATAATCACGTAGCTACATTCCTCAAAGGACGAGCGCGGTGACGTCACGCATGATA---------//   -300bp   //-----------------------------------------CATCTCTATCGGCATTACAAGATG     -349(-397,+48)
AGTGCAGCAAAGTATCCACGTTTAATGTAATATAATCACAGGGTTAGGGTTAGGGTTAGGGTTAGGGTTAGG-----------------------//   -300bp   //-------------------------------------TGCATCTCTATCGGCATTACAAGATG     -362(-393,+31)
AGTGCAGCAAAGTATCCACGTTTAATGTAATATAATCACAGCACATGTACACAGTATCACA----------------------------------//   -300bp   //-------------------------------GTACATGATGCATCTCTATCGGCATTACAAGATG     -365(-383,+18)
AGTGCAGCAAAGTATCCACGTTTAATGTAATATAATCACAGCAGCATCTCTATCGGCATTATCA-------------------------------//   -300bp   //-------------------------------------GATGCATCTCTATCGGCATTACAAGATG     -368(-388,+20)
AGTGCAGCAAAGTATCCACGTTTAATGTAATATAATCACAGATAATGTAATATAATC--------------------------------------//   -300bp   //----------------------------------ACATGATGCATCTCTATCGGCATTACAAGATG     -372(-387,+15)
AGTGCAGCAAAGTATCCACGTTTAATGTAATATAATCACTAGACGCATGCATAGAC---------------------------------------//   -300bp   //------------------------------------TGATGCATCTCTATCGGCATTACAAGATG     -374(-392,+18)
AGTGCAGCAAAGTATCCACGTTTAATGTAATATAATCACAGCATCTCTATGCATCTC--------------------------------------//   -300bp   //-------------------------------------GATGCATCTCTATCGGCATTACAAGATG     -375(-389,+14)
AGTGCAGCAAAGTATCCACGTTTAATGTAATATAATCACAGCTGGGTTAGGGTTAGGGGTT----------------------------------//   -300bp   //-----------------------------------ATGATGCATCTCTATCGGCATTACAAGATG     -379(-388,+19)
AGTGCAGCAAAGTATCCACGTTTAATGTAATATAATCACTAGACTATAG----------------------------------------------//   -300bp   //----------------------------------CATGATGCATCTCTATCGGCATTACAAGATG     -380(-390,+10)
AGTGCAGCAAAGTATCCACGTTTAATGTAATATAATCACTAGACTATAGTATC------------------------------------------//   -300bp   //------------------------------------TGATGCATCTCTATCGGCATTACAAGATG     -380(-392,+12)
AGTGCAGCAAAGTATCCACGTTTAATGTAATATAATCACAGCATATATA----------------------------------------------//   -300bp   //-----------------------------------ATGATGCATCTCTATCGGCATTACAAGATG     -381(-387,+6)
AGTGCAGCAAAGTATCCACGTTTAATGTAATATAATCACAGCATAATCACA--------------------------------------------//   -300bp   //--------------------------------------ATGCATCTCTATCGGCATTACAAGATG     -382(-390,+8)
AGTGCAGCAAAGTATCCACGTTTAATGTAATATAATCACAGCATA--------------------------------------------------//   -300bp   //-----------------------------------ATGATGCATCTCTATCGGCATTACAAGATG     -385(-387,+2)
AGTGCAGCAAAGTATCCACGTTTAATGTAATATAATCACTAGACTA-------------------------------------------------//   -300bp   //-------------------------------------GATGCATCTCTATCGGCATTACAAGATG     -386(-393,+7)
AGTGCAGCAAAGTATCCACGTTTAATGTAATATAATCACAGCA----------------------------------------------------//   -300bp   //----------------------------------CATGATGCATCTCTATCGGCATTACAAGATG     -386
AGTGCAGCAAAGTATCCACGTTTAATGTAATATAATCACAGCTC---------------------------------------------------//   -300bp   //------------------------------------TGATGCATCTCTATCGGCATTACAAGATG     -387(-389,+2)
AGTGCAGCAAAGTATCCACGTTTAATGTGATATAATCACTAGACTATA-----------------------------------------------//   -300bp   //----------------------------------------GCATCTCTATCGGCATTACAAGATG     -387(-407,+20)
AGTGCAGCAAAGTATCCACGTTTAATGTAATATAATCACAGAGA---------------------------------------------------//   -300bp   //-------------------------------------GATGCATCTCTATCGGCATTACAAGATG     -388(-391,+3)
AGTGCAGCAAAGTATCCACGTTTAATGTAATATAATCACTAGACT--------------------------------------------------//   -300bp   //--------------------------------------ATGCATCTCTATCGGCATTACAAGATG     -388(-394,+6)
AGTGCAGCAAAGTATCCACGTTTAATGTAATATAATCACAGCA----------------------------------------------------//   -300bp   //------------------------------------TGATGCATCTCTATCGGCATTACAAGATG     -388
AGTGCAGCAAAGTATCCACGTTTAATGTAATATAATCACACG-----------------------------------------------------//   -300bp   //------------------------------------TGATGCATCTCTATCGGCATTACAAGATG     -389(-391,+2)
AGTGCAGCAAAGTATCCACGTTTAATGTAATATAATCACA-------------------------------------------------------//   -300bp   //----------------------------------CATGATGCATCTCTATCGGCATTACAAGATG     -389
AGTGCAGCAAAGTATCCACGTTTAATGTAATATAATCACAG------------------------------------------------------//   -300bp   //-----------------------------------ATGATGCATCTCTATCGGCATTACAAGATG     -389
AGTGCAGCAAAGTATCCACGTTTAATGTAATATAATCACTA------------------------------------------------------//   -300bp   //-------------------------------------GATGCATCTCTATCGGCATTACAAGATG     -391(-393,+2)
AGTGCAGCAAAGTATCCACGTTTAATGTAATATAATCACA-------------------------------------------------------//   -300bp   //------------------------------------TGATGCATCTCTATCGGCATTACAAGATG     -391
AGTGCAGCAAAGTATCCACGTTTAATGTAATATAATCACAG------------------------------------------------------//   -300bp   //--------------------------------------ATGCATCTCTATCGGCATTACAAGATG     -392
AGTGCAGCAAAGTATCCACGTTTAATGTAATATAATCACA-------------------------------------------------------//   -300bp   //---------------------------------------TGCATCTCTATCGGCATTACAAGATG     -394
AGTGCAGCAAAGTATCCACGTTTAATGTAATATAATCACAGCATCTCTATCGGCATC--------------------------------------//   -300bp   //---------------------------------------------------------ACAAGATG     -395(-490,+14)
AGTGCAGCAAAGTATCCACGTTTAATGTAATATAATCACAGC-----------------------------------------------------//   -300bp   //-----------------------------------------------TCTATCGGCATTACAAGATG     -398
AGTGCAGCAAAGTATCCACGTTTAATG--------------------------------------------------------------------//   -300bp   //--------------------------------------ATGCATCTCTATCGGCATTACAAGATG     -406
AGA--------------------------------------------------------------------------------------------//   -300bp   //---------------------------------TACATGATGCATCTCTATCGGCATTACAAGATG     -424(-425,+1)
AGTGCAGCAAAGTATCCACGTTTAATGTAATATAATCACTAGACA--------------------------------------------------//   -300bp   //----------------------------------------//-40bp//--CCTCGAGCACGAGC     -471(-477,+6)
AGTGCAGCAAAGTATCCACGTTTAATGTAATATAATCAC----------GTAGCTACATTACCTTCAAAGGACGAGCGCGGTGACGTCACG----//   -300bp   //--------------------------------------ATGCATCTCTATCGGCATTACAAGATG     -341, [5’]-10

AGTGCAGCAAAATATC-------------------------------------------------------------------------------//-70bp, +230bp//GGAATATGTCACACCGCATGCATCTGCATCTCTATCGGCA--------------TTACAAGATG     [5’]-149, [3’]-16

AGTGCAGCAAAGTATCCACGTTTAATGTAATATAATCACGGCGGTTCACGTAGCTACATTACCTTCAAAGGACGAGCGCGGTGACGTCACGCGCG//   +300bp   //GGAATATGTCACACCGCATGCATCTGCCCACGTACATGATGCATCTCTATCGGCATTACAAGATG     [5’] 2m                    x4
AAAGTATCCACGTTTAATGTAATATAATCACTAGACTATAGCAGTTCACGTAGCTACATTACCTTCAAAGGACGAGCGCGGTGACGTCACGCGCG//   +300bp   //GGAATATGTCACACCGCATGCATCTGCCCACGTACATGATGCATCTCTATCGGCATTACAAGATG     [5’]+8                      x7
AAAGTATCCACGTTTAATGTAATATAATCACTAGACTATAGCAGCTCACGTAGCTACATTACCTTCAAAGGACGAGCGCGGTGACGTCACGCGCG//   +300bp   //GGAATATGTCACACCGCATGCATCTGCCCACGTACATGATGCATCTCTATCGGCATTACAAGATG     [5’]+8(-6,+14)
GCAGCAAAGTATCCACGTTTAATGTAATCACTAGACTATAGCAGTTCACGTAGCTACATTACCTTCAAAGGACGAGCGCGGTGACGTCACGCGCG//   +300bp   //GGAATATGTCACACCGCATGCATCTGCCCACGTACATGATGCATCTCTATCGGCATTACAAGATG     [5’]+3(-8,+11)
TGCAGCAAAGTATCCACGTTTAATGTAATATAATCACAAATTATATTACGTAGCTACATTACCTTCAAAGGACGAGCGCGGTGACGTCACGCGCG//   +300bp   //GGAATATGTCACACCGCATGCATCTGCCCACGTACATGATGCATCTCTATCGGCATTACAAGATG     [5’]+2(-9,+11)
AGTGCAGCAAAGTATCCACGTTTAATGTAATATAATCAC----------GTAGCTACATTACCTTCAAAGGACGAGCGCGGTGACGTCACGCGCG//   +300bp   //GGAATATGTCACACCGCATGCATCTGCCCACGTACATGATGCATCTCTATCGGCATTACAAGATG     [5’]-10
AGTGCAGCAAAGTATCCACGTTTAATGTAATATAATCACAGCTACA-----------ATTACCTTCAAAGGACGAGCGCGGTGACGTCACGCGCG//   +300bp   //GGAATATGTCACACCGCATGCATCTGCCCACGTACATGATGCATCTCTATCGGCATTACAAGATG     [5’]-11(-15,+4)
AAAGTATCCACGTTTAATGTAATATAATCACTAGACTATAGCAGTTCACGTAGCTACATTACCTTCAAAGGACGAGCGCGGTGACGTCACGCGCG//   +300bp   //GGAATATGTCACACCGCATGCATCT---------------------CTATCGGCATTACAAGATG     [5’]+8       [3’](-21)
AAAGTATCCACGTTTAATGTAATATAATCACTAGACTATAGCAGTTCACGTAGCTACATTACCTTCAAAGGACGAGCGCGGTGACGTCACGCGCG//   +300bp   //GGAATATGTCACACCGCATGCATCTGCCCACGT--ATGATGCATCTCTATCGGCATTACAAGATG     [5’]+8       [3’]-2         x2
TGCAGCAAAGTATCCACGTTTAATGTAATATAATCACAGCAGAGTTCACGTAGCTACATTACCTTCAAAGGACGAGCGCGGTGACGTCACGCGCG//   +300bp   //GGAATATGTCACACCGCATGCATCTGCCCACGT--ATGATGCATCTCTATCGGCATTACAAGATG     [5’]+2       [3’]-2
AGTGCAGCAAAGTATCCACGTTTAATGTAATATAATCACAG---------TAGCTACATTACCTTCAAAGGACGAGCGCGGTGACGTCACGCGCG//   +300bp   //GGAATATGTCACACCGCATGCATCTGCCCA------TGATGCATCTCTATCGGCATTACAAGATG     [5’]-9       [3’]-6
AGTGCAGCAAAGTATCCACGTTTAATGTAATATAATCACAGCA-----CGTAGCTACATTACCTTCAAAGGACGAGCGCGGTGACGTCACGCGCG//   +300bp   //GGAATATGTCACACCGCATGCATCTGCCCACATCTGCC---CATCTCTATCGGCATTACAAGATG     [5’]-5       [3’]-3(-10,+7)
AGTGCAGCAAAGTATCCACGTTTAATGTAATATAATCACAGCA-----CGTAGCTACATTACCTTCAAAGGACGAGCGCGGTGACGTCACGCGCG//   +300bp   //GGAATATGTCACACCGCATGCATCTGCCCA------TGATGCATCTCTATCGGCATTACAAGATG     [5’]-5       [3’]-6
AGTGCAGCAAAGTATCCACGTTTAATGTAATATAATCACAG---------TAGCTACATTACCTTCAAAGGACGAGCGCGGTGACGTCACGCGCG//   +300bp   //GGAATATGTCACACCGCATGCATCTGCCCACG------ATGCATCTCTATCGGCATTACAAGATG     [5’]-9       [3’]-6
AGTGCAGCAAAGTATCCACGTTTAATGTAATATAATCAC----------GTAGCTACATTACCTTCAAAGGACGAGCGCGGTGACGTCACGCGCG//   +300bp   //GGAATATGTCACACCGCATGCATCTGCCCACGTACCATGATGCATCTCTATCGGCATTACAAGAT     [5’]-10      [3’]+1

AGTGCAGCAAAGTATCCACGTTTAATGTAATATAATCACAGCAGTTCACGTAGCTACATTACCTTCAAAGGACGAGCGCGGTGACGTCACGCGCG//   +300bp   //GGAATATGTCACACCGCATGCATCTGCCCACGCATCTGCCCATGATGCATCTCTATCGGCATTAC     [3’]+6(-2,+8)
AGTGCAGCAAAGTATCCACGTTTAATGTAATATAATCACAGCAGTTCACGTAGCTACATTACCTTCAAAGGACGAGCGCGGTGACGTCACGCGCG//   +300bp   //GGAATATGTCACACCGCATGCATCTGCCCACGTATACATGATGCATCTCTATCGGCATTACAAGA     [3’]+2
AGTGCAGCAAAGTATCCACGTTTAATGTAATATAATCACAGCAGTTCACGTAGCTACATTACCTTCAAAGGACGAGCGCGGTGACGTCACGCGCG//   +300bp   //GGAATATGTCACACCGCATGCATCTGCCCA------TGATGCATCTCTATCGGCATTACAAGATG     [3’]-6
AGTGCAGCAAAGTATCCACGTTTAATGTAATATAATCACAGCAGTTCACGTAGCTACATTACCTTCAAAGGACGAGCGCGGTGACGTCACGCGCG//   +300bp   //GGAATATGTCACACCGCATGCATCTGCCCA-------------TCTCTATCGGCATTACAAGATG     [3’]-13
AGTGCAGCAAAGTATCCACGTTTAATGTAATATAATCACAGCAGTTCACGTAGCTACATTACCTTCAAAGGACGAGCGCGGTGACGTCACGCGCG//   +300bp   //GGAATATGTCACACCGCATGCATCT---------------------CTATCGGCATTACAAGATG     [3’]-21
```

Figure S1.

H

egfp5TAL(12.5pg) Mutation Frequency = 11.6% (5/43)

GTGAGCAAGGGCGAGGAGCTGTTCACCGGGGTGGTGCCCATCCTGGTCGAGCTGGACGGCGACGTA	wt	x38
GTGAGCAAGGGCGAGGAGCTGTTCACCGGG-TGGTGCCCATCCTGGTCGAGCTGGACGGCGACGTA	-1	
GTGAGCAAGGGCGAG------------------------------------CTGGACGGCGACGTA	-36	
GTGAGCAAGGGCGAGGAGCTGTTCACCGGGGTGGGTGCCCATCCTGGTCGAGCTGGACGGCGACGT	+1	
GTGAGCAAGGGCGAGGAGCTGTTCACCGGGGTGGTGTGCCCATCCTGGTCGAGCTGGACGGCGACG	+2	
GTGAGCAAGGGCGAGGAGCTGTTCACCGAGGTGGTGGTGCCCATCCTGGTCGAGCTGGACGGCGAC	+3	

egfp3TAL(12.5pg) Mutation Frequency = 21.4% (9/42)

CTGAGCACCCAGTCCGCCCTGAGCAAAGACCCCAACGAGAAGCGCGATCACATGGTCCTGCTGGAG	wt	x33
CTGAGCATCCAGTCCGCCCTGAGCAAAGACCCCAACGAGAAGCGCGATCACATGGTCCTGCTGGAG	1m	
CTGAGCACCCAGTCCGCCCTGAGCAAAGACCCCAACG--AAGCGCGATCACATGGTCCTGCTGGAG	-2	
CTGAGCACCCAGTCCGCCCTGAGCAAAGAAACGAC--AGAAGCGCGATCACATGGTCCTGCTGGAG	-2(-8,+6)	
CTGAGCACCCAGTCCGCCCTGAGCAAAGACCCCCAAC----GCGCGATCACATGGTCCTGCTGGAG	-4(-12,+8)	
CTGAGCACCCAGTCCGCCCTGAGCAA---------------GCGCGATCACATGGTCCTGCTGGAG	-15	
CTGAGCACCCAGTCCGCCCTGAGCAAAGA------------------TCACATGGTCCTGCTGGAG	-18	
CTGAGCACCCAGTCCGCCCC----------------------CGCGATCACATGGTCCTGCTGGAG	-22(-23,+1)	
CTGAGCACCCAGTCCGCCCTGAGCAAAGACCCCAACGAGAGAGCGCGATCACATGGTCCTGCTGGA	+1	
CTGAGCACCCAGTCCGCCCTGAGCAAAGACCCCCAACGAGAAGCGCGATCACATGGTCCTGCTGGA	+1	

Figure S1.

sqt5TAL(25pg) Mutation Frequency = 17.8% (8/45)

CTGCAAGTTTCTATAAGTGAACTTAGATGACCGGCCAGCACTCATGACATTCACTTTCCAGAGGA	wt	x37
CTGCAAGTTTCTATAAATGAACTTAGATGACCGGCCAGCACTCATGACATTCACTTTCCAGAGGA	1m	
CTGCAAGTTTCTATAAGCGAACTTAGATGACCGGCCAGCACTCATGACATTCACTTTCCAGAGGA	1m	
CTGCAAGTTTCTATAAGTGAACTTAGGTGACCGGCCAGCACTCATGACATTCACTTTCCAGAGGA	1m	
CTGCAAGTTTCTATAAGTGAACTTAGATGACCGGCCAGCACCCATGACATTCACTTTCCAGAGGA	1m	
CTGCAAGTTTCTATAAGTGAACTTAGATG---------//---------TCTACCGGACACTTCT	-288	
CTGCAAGTTTCTATAAGTGAACTTAGATGACCGGGCCAGCACTCATGACATTCACTTTCCAGAGG	+1	x3

sqt3TAL(25pg) Mutation Frequency = 12.2% (5/41)

ATGCTATTATTGAAAGCTTTGCGTGTTTGCCTTATCTGTAAATAGTAGAGTATGTAAATTACCAA	wt	x36
ATGCTATTATTGAAAGCCTTGCGTGTTTGCCTTATCTGNAAATAGTAGAGTATGNAAATTACCAA	1m	x2
ATGCTATTATTGAAAGCTTTGCGTGTTTGC-TTATCTGTAAATAGTAGAGTATGTAAATTACCAA	-1	
ATGCTATTATTGAAAGCTTTGCGTGTTTG-----TCTGTAACTAGTAGAGTATGTAAATTACCAA	-5,1m	
ATGCTATTATTGAAAGCTTTGCGTGTTTGCCTTCTTATCTGTAAATAGTAGAGTATGTAAATTAC	+3	

sqtZFN2(25pg) Mutation Frequency = 11.1% (5/45)

GCACGGGAAACCTTGCTGGATTTCAAGAGACCCTCAGGAATAAAATGCGA	wt	x40
GCACGGGAAGCCTTGCTGGATTTCAAGAGACCCTCAGGAATAAAATGCGA	1m	
GCACGGGAAACCTTGCTGGATTTCGAGAGACCCTCAGGAATAAAATGCGA	1m	
GCACGGGAAACCTTGCTGGATTTCAGGAGACCCTCAGGAATAAAATGCGA	1m	
GCACGGGAAACCTTGCTGGA--------GACCCTCAGGAATAAAATGCGA	-8	
GCACGGGAAACC--------------------CTCAGGAATAAAATGCGA	-20	

Figure S1.

J

cyc5TAL(12.5pg) Mutation Frequency = 48.8% (21/43)

ATCCACGTTTAATGTAATATAATCACAGCAGTTCACGTAGCTACATTACCTTCAAAGGAC	wt	x22
ATCCACGTTTAATGTAATATAATCACAGCAGTTCACGTAGCTACATCACCTTCAAAGGAC	1m	
ATCCACGTTTAATGTAATATAATCACAGCA-TTCACGTAGCTACATTACCTTCAAAGGAC	-1	
ATCCACGTTTAATGTAATATAATCACA-----TCACGTAGCTACATTACCTTCAAAGGAC	-5	
ATCCACGTTTAATGTAATATAATCACAG---------TAGCTACATTACCTTCAAAGGAC	-9	
ATCCACGTTTAATGTAATATAATCAC----------GTAGCTACATTACCTTCAAAGGAC	-10	x6
ATCCACGTAGCTAT------------------------------ATTACCTTCAAAGGAC	-30(-36,+6)	
ATCCACGTTTAATGTAATATAATCACTAGACTATAGCAGTTCACGTAGCTACATTACCTT	+8	x10

cyc3TAL(12.5pg) Mutation Frequency = 61.3% (19/31)

ATATGTCACACCGCATGCATCTGCCCACGTACATGATGCATCTCTATCGGCATTACAAGA	wt	x12
ATATGTCACACCACATGCATCTGCCCACGTACATGATGCATCTCTATCGGCATTACAAGA	1m	
ATATGTCACACCGCATGCATCTGCCCACGTAAATGATGCATCTCTATCGGCATTACAAGA	1m	
ATATGTCACACCGCATGCATCTGCCCACGT-CGTGATGCATCTCTATCGGCATTACAAGA	-1(-3,+2)	
ATATGTCACACCGCATGCATCTGCCC----ACGTGATGCATCTCTATCGGCATTACAAGA	-4	
ATATGTCACACCGCATGCATCTGCCCAC-----TGATGCATCTCTATCGGCATTACAAGA	-5	
ATATGTCACACCGCATGCATCTGCCCACGC-----ATGCATCTCTATCGGCATTACAAGA	-5(-6 +1)	
ATATGTCACACCGCATGCATCTGCCCACG------ATGCATCTCTATCGGCATTACAAGA	-6	x2
ATATGTCACACCGCATGCATCTGCCCA---------TGCATCTCTATCGGCATTACAAGA	-9	
ATATGTCACACCGCATGCATCTGCCCACG---------CATCTCTATCGGCATTACAAGA	-9	
ATATGTCACACCGCATGCATCTGCCCACT-----------TCTCTATCGGCATTACAAGA	-11(-12,+1)	
ATATGTCACACCGCATGCATCTG------------ATGCATCTCTATCGGCATTACAAGA	-12	
ATATGTCACACCGCATGCATCTGCCCA-------------TCTCTATCGGCATTACAAGA	-13	
ATATGTCACACCGCATGTATCTGC---------------ATCTCTATCGGCATTACAAGA	-15, 1m	
ATATGTCACACCGCATGCATCTGCCCACGGC--------------ATCGGCATTACAAGA	-14(-16,+2)	
ATATGTCACACCGCATGCATCTGCCCA------TGATGCATCTCTATCGGCATTACAAGA	-16	
ATATGTCACACCGCATGCATCTGCCCACG--------------------GCATTACAAGA	-20	
ATATGTCACACCGCATGCATCT---------------------CTATCGGCATTACAAGA	-21	
ATATGTCACACCGCATGCATCTGCCCACGTACTACATGATGCATCTCTATCGGCATTACA	+3	

Figure S1.

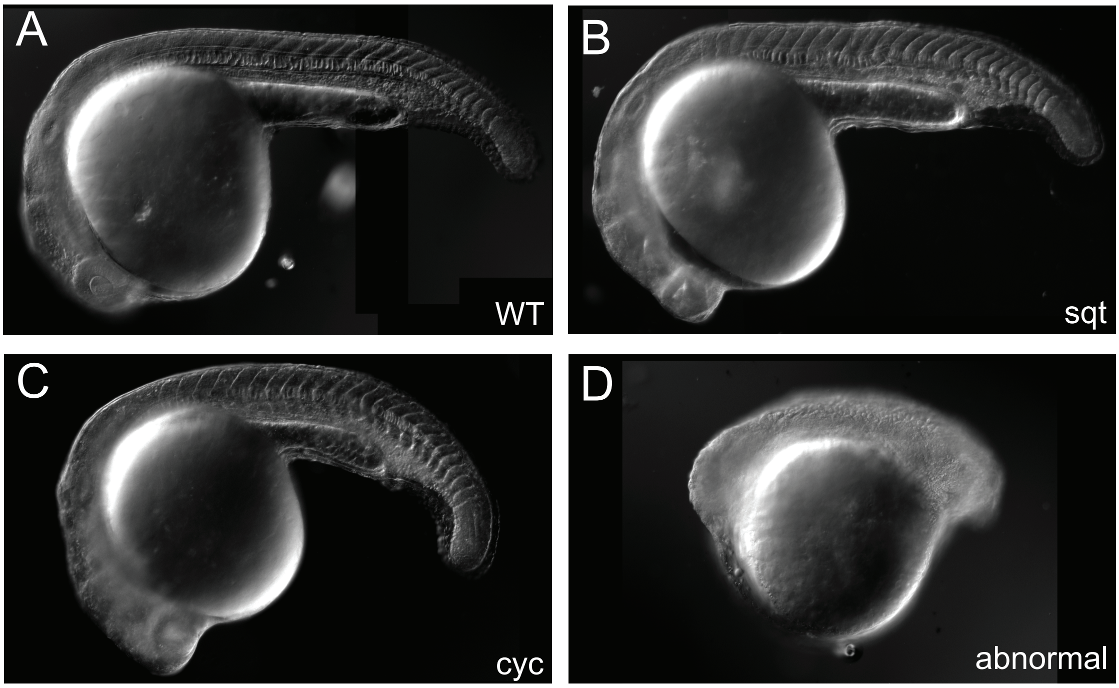

Figure S2. Representative phenotypes observed at 24 h in embryos injected with *sqt* or *cyc* TALENs. At high doses, the proportion of abnormal embryos increases.

A

```
                                     cyc5TAL Left Arm
49427780  WT         CCACGTTTAATGTAATATAATCACAGCAGTTCACGTAGCTACATTACCTTCAAAGGA
          F0-1A      CCACGTTTAATGTAATATAATCACAGCATCTCT------------------------
          F0-1B      CCACGTTTAATGTAATATAATCACAGCACAT--------------------------
          F0-2,7,9A  CCACGTTTAATGTAATATAATCACAGCATCTG-------------------------
          F0-3       CCACGTTTAATGTAATATAATCACAG-------------------------------
          F0-4       CCACGTTTAATGTAATATAATCACAGTGGTGGTGG----------------------
          F0-5       CCACGTTTAATGTAATATAATCACAGCAGT---------------------------
          F0-6       CCACGTTTAATGTAATATAATCACA--------------------------------
          F0-8       CCACGTTTAATGTAATATAATCACAGCATCTAACAGTAATATAATGATGCA------
          F0-9B      CCACGTTTAATGTAATATAATCACAATCA----------------------------
--------//340 bp//--------
                  cyc3TAL Right Arm
ACGTACATGATGCATCTCTATCGGCATTACAAGATGAACCA  WT                   49428229
-------------ATCTCTATCGGCATTACAAGATGAACCA  F0-1A      (-394 bp/+5 bp)
----------TGCATCTCTATCGGCATTACAAGATGAACCA  F0-1B      (-391 bp/+3 bp)
------ATGATGCATCTCTATCGGCATTACAAGATGAACCA  F0-2,7,9A  (-387 bp/+4 bp)
---------ATGCATCTCTATCGGCATTACAAGATGAACCA  F0-3       (-392 bp)
-----------------------------------------  F0-4       (-467 bp/+9 bp)
---------------CTCTATCGGCATTACAAGATGAACCA  F0-5       (-394 bp)
----------TGCATCTCTATCGGCATTACAAGATGAACCA  F0-6       (-394 bp)
--------------TCTCTATCGGCATTACAAGATGAACCA  F0-8       (-397 bp/+23 bp)
---------ATGCATCTCTATCGGCATTACAAGATGAACCA  F0-9B      (-394 bp/+4 bp)
```

B

```
                         cyc5TAL Left Arm                    cyc5TAL Right Arm
WT      CAAAGTGCAGCAAAGTATCCACGTTTAATGTAATATAATCACAGCAGTTCACGTAGCTACATTACCT
F0-10A  CAAAGTGCAGCAAAGTATCCACGTTTAATGTAATATAATCACAG-----------------------

WT      TCAAAGGACGAGCGCGGTGACGTCACGCGCGGGCGCAAGGTGTTTTAATTGTTACCACACGAATAGA
F0-10A  -------------------------------------------------------------------

WT      TGATGAGCTGTCACGGCATGTCAATTTCCTTTAATGTCCTCTGCTACTTGTTAAATATGCGGGGGTC
F0-10A  -------------------------------------------------------------------

WT      CGACTCGAGGGAGGCCCTATATAAAGCGCCCGGGACTCCCGAGCTGTCATTCATTCTGACCGCGCAT
F0-10A  ------------------------------TGTTCAGATGCATGCGGTGTGACATATTCCTCAAGCT

WT      CATGCACGCGCTCGGAGTCGCGCGCCTGGCTTGCTACTGCCAGCTGCTGCTGCTCGGAGTGTTCGGA
F0-10A  GTTATAATTATTGTGATGATAACGCGTGTGCTTTCCGAACACTCCGAGCAGCAGCAGCTGGCAGTAG

                                                           cyc3TAL Left Arm
WT      AAGCACACGCGTTATCGTCACAATAATTATAACAGCTTGAGGAATATGTCACACCGCATGCATCTGC
F0-10A  CAGGCCAGGCGCGCGACTCCGAGCGCGTGCATGATGCGCGGTCAGAATGAATGACAGCTCGGGAGTC

                          cyc3TAL Right Arm
WT      CCACGTACATGATGCATCTCTATCGGCATTACAAGATGAACCAGACGCGCATACCCGCGGAGAGCCT
F0-10A  CCG         TGATGCATCTCTATCGGCATTACAAGATGAACCAGACGCGCATACCCGCGGAGAGCCT
          AGTACCCATGCATC

WT      CGAGCACGAGCACGCAGACACAATCAGGAGCATCATGTCT
F0-10A  CGAGCACGAGCACGCAGACACAATCAGGAGCATCATGTCT
```

Figure S3.

A. Alignment of *cyc* sequences showing TSS deletions in embryos from F0 founders, compared to wild type *cyc*. Insertions are indicated in red, and gaps are shown by dashed lines. Letter suffixes (e.g., 1A and 1B) represent different alleles from the same founder. Eight founders injected with *cyc* TALENs transmitted complete deletion of the intervening sequences, some of which also show insertion events. Founder F0-4, shows a larger deletion that extends beyond the 3'end of cyc3TAL target site. Genomic coordinates on chromosome 12 for wild type *cyc* are indicated for the regions shown.

B. Alignment of *cyc* sequence of F0-10 to wild type *cyc* shows a deletion (dashed lines), accompanied by an inversion (yellow highlight) and insertion (red font).

A

```
                              sqt5TAL Left Arm
19838700  WT     AAGACTGCAAGTTTCTATAAGTGAACTTAGATGACCGGCCAGCACTCATGACATTC
          F0-1   AAGACTGCAAGTTTCTATAAGTGAACTGA---------------------------
          F0-2   AAGACTGCAAGTTTCTATAAGTGAACTTAGATGACTGTAAATGAA-----------
          F0-3   AAGACTGCAAGTTTCTATAAGTGAACTTAGATGACT--------------------
          F0-4   AAGACTGCAAGTTTCTATAAGTGAACTTAGATGACTT-------------------
          F0-5   AAGACTGCAAGTTTCTATAAGTGAACTTAGAT------------------------
          F0-6   AAGACTGCAAGTTTCTATAAGTGAACTTAGATGATCTGTAAATAG-----------

--------//2143 bp//--------

                                         sqt3TAL Right Arm
AGCTTTGCGTGTTTGCCTTATCTGTAAATAGTAGAGTATGTAAATTACCAAAT  WT     19840933
----------------CTTATCTGTAAATAGTAGAGTATGTAAATTACCAAAT  F0-1
---------------------CTGTAAATAGTAGAGTATGTAAATTACCAAAT  F0-2
-----------------TTATCTGTAAATAGTAGAGTATGTAAATTACCAAAT  F0-3
--------------------TCTGTAAATAGTAGAGTATGTAAATTACCAAAT  F0-4
-------------------------AAATAGTAGAGTATGTAAATTACCAAAT  F0-5
------------------TATCTGTAAATAGTAGAGTATGTAAATTACCAAAT  F0-6
```

B

```
                              sqt5TAL Left Arm
19838700  WT     AAGACTGCAAGTTTCTATAAGTGAACTTAGATGACCGGCCAGCACTCATGACATTC
          F0-1   AAGACTGCAAGTTTCTATAAGTGAACTTAGATGACTTAGATGA-------------
          F0-2   AAGACTGCAAGTTTCTATAAGTGAACTCATGAGTTCAAGTGAA-------------

ACTTTCCAGAGGACGGTGTAAGAGAAAGAGCTCAGAGACTTTATTTCAATAACTGCGTGTGGATTATTACC
-----------------------------------------------------------------------
-----------------------------------------------------------------------

TTGATTTGACATGTTTTCCTGCGGGCTCCTGAGCGTAGTTTTGGCCCTCGCGGTCGGCCTGGTGAGCTGCA
-----------------------------------------------------------------------
--------------TTTCCTGCGGGCTCCTGAGCGTAGTTTTGGCCCTCGCGGTCGGCCTGGTGAGCTGCA

       sqtZFN2 Left Arm       sqtZFN2 Right Arm
CGGGAAACCTTGCTGGATTTCAAGAGACCCTCAGGAATAAA  WT    19838938
------------------------------TCAGGAATAAA  F0-1
CGGGAAACCTTGCTGGATTTCAAGAGACCCTCAGGAATAAA  F0-2
```

Figure S4.

A. Alignment of *sqt* sequences showing the whole-locus and TSS deletions in embryos from F0 founders, compared to wild type *sqt*. Insertions are highlighted in red font, and gaps are indicated with dashed lines. All 6 founders for sqt5TAL/sqt3TAL showed embryos with the 2.1 kb whole locus deletion.

B. For sqt5TAL/sqtZFN2, founder 1 (F0-1) showed embryos with the intervening sequences excised, whereas founder 2 (F0-2) sequences indicate that only sqt5TAL was active. Genomic coordinates on chromosome 21 for wild type *sqt* are indicated for the regions shown. TALEN and ZFN target sites are indicated.

A

```
                                 sqt5TAL Left Arm
19838700     WT  AAGACTGCAAGTTTCTATAAGTGAACTTAGATGACCGGCCAGCACTCATGACATTC
           sg27  AAGACTGCAAGTTTCTATAAGTGAACTCATGAGTTCAAGTGAA-------------

ACTTTCCAGAGGACGGTGTAAGAGAAAGAGCTCAGAGACTTTATTTCAATAACTGCGTGTGGATTATTACC
-----------------------------------------------------------------------

TTGATTTGACATGTTTTCCTGCGGGCTCCTGAGCGTAGTTTTGGCCCTCGCGGTCGGCCTGGTGAGCTGCA
--------------TTTCCTGCGGGCTCCTGAGCGTAGTTTTGGCCCTCGCGGTCGGCCTGGTGAGCTGCA

         sqtZFN2 Left Arm    sqtZFN2 Right Arm
CGGGAAACCTTGCTGGATTTCAAGAGACCCTCAGGAATAAA       19838938
CGGGAAACCTTGCTGGATTTCAAGAGACCCTCAGGAATAAA
```

B

```
                                 sqt5TAL Left Arm
19838700     WT  AAGACTGCAAGTTTCTATAAGTGAACTTAGATGACCGGCCAGCACTCATGACATTC
           sg32  AAGACTGCAAGTTTCTATAAGTGAACTTAGATGATCTGTAAATAG-----------

--------//2143 bp//--------

                                          sqt3TAL Right Arm
AGCTTTGCGTGTTTGCCTTATCTGTAAATAGTAGAGTATGTAAATTACCAAAT       19840933
------------------TATCTGTAAATAGTAGAGTATGTAAATTACCAAAT
```

C

```
                          sqtZFN1 Left Arm      sqtZFN1 Right Arm
19839873     WT  TTTATCACGCATCTACACC----GGAGTGTGAGAGAAGCCCC      19839910
            sg7  TTTATCACGCATCTACACCGGCCGGAGTGTGAGAGAAGCCCC
```

Figure S5. Alignment of sqt sequences showing the *sqt*^{sg27} TSS deletion **(A)**, *sqt*^{sg32} whole-locus deletion **(B)**, and *sqt*^{sg7} ZFN **(C)** mutations in comparison to wild type *sqt*. Insertions are highlighted in red font, and gaps are indicated with dashed lines. TALEN and ZFN target sites are indicated.

Table S1. List of target sites for TALENs and ZFNs

Target Gene	Targeting nuclease	Target site sequences (5' to 3')
sqt	sqt5TAL	GCAAGTTTCTATAAGTgaacttagatgaccggccagcactcatgacATTCACTTTCCAGAGG
	sqt3TAL	GCTATTATTGAAAGCTttgcgtgtttgccttatctgtaaatagtAGAGTATGTAAATTACC
	sqtZFN1	TCACGCATCTACacctgaGTGTGAGAGAAG
	sqtZFN2	CCTTGCTGGATTtcaagaGACCCTCAGGAA
egfp	egfp5TAL	GAGCAAGGGCGAGGAGCTgttcaccggggtggtgcccatcctggtcgAGCTGGACGGCGACGT
	egfp3TAL	GAGCACCCAGTCCGCCCTgagcaaagaccccaacgagaagcgcgATCACATGGTCCTGCTGG
cyc	cyc5TAL	CCACGTTTAATGTAATATAATcacagcagttcacgtAGCTACATTACCTTCAAAGG
	cyc3TAL	ATGTCACACCGCATGCATCTgcccacgtacatgatgcATCTCTATCGGCATTACAAG

Binding sites are shown in uppercase, and spacer sequences in lowercase.

Table S2. List of primers for genotyping, sequencing, and T7E1 assays.

Primer Labels and Sequences

Primer Labels	Sequence 5'-3'
sqt5TF	TGTATGCCTTTATGGATCACAGG
sqt5TR	CATGTCAAATCAAGGTAATAATCCAC
sqt3TF	GTTTCCTGCACTGAGGCACCTG
sqt3TR	CCTTATTCATTCACTCCCTCGTG
sqtZFN2F	TTTCCAGAGGACGGTGTAAGA
sqtZFN2R	TTAGCGACGAGGCTCAAGA
cyc5G1F	GAATAAATACTGCTTGCAAG
cyc5G1R	TTGACATGCCGTGACAGCTC
cyc5G2F	CTGGCTTGCTACTGCCAG
cyc5G2R	AGACATGATGCTCCTGATTG
egfpT1F	CCTATCAGAAACTGCAGTATCTG
egfpT1R	GATGAACTTCAGGGTCAGCTTGC
egfpT2F	CACAACATCGAGGACGGCAG
egfpT2R	CTCTACAAATGTGGTATGGCTG

Primer combinations used to amplify nuclease-targeted regions for sequencing and T7E1 assays.

Nuclease(s) used	Primer Combinations		Expected Amplicon Size	
	Forward	Reverse	WT	Mutant*
sqt5TAL	sqt5TF	sqt5TR	203bp	
sqt3TAL	sqt3TF	sqt3TR	179bp	
sqtZFN2	sqtZFN2F	sqtZFN2R	357bp	
sqt5TAL + sqt3TAL	sqt5TF	sqt3TR	2378bp	217bp
sqt5TAL + sqtZFN2	sqt5TF	sqtZFN2R	478bp	294bp
cyc5TAL	cyc5G1F	cyc5G1R	244bp	
cyc3TAL	cyc5G2F	cyc5G2R	216bp	
cyc5TAL + cyc3TAL	cyc5G1F	cyc5G2R	595bp	209bp
egfp5TAL	egfpT1F	egfpT1R	227bp	
egfp3TAL	egfpT2F	egfpT2R	264bp	
egfp5TAL + egfp3TAL	egfpT1F	egfpT2R	854bp	256bp

*Expected mutant amplicon size if both nuclease pairs cut precisely in the middle of the target site.

Table S3. List of primers to detect expression of *sqt, rnf180, htr1ab, eif4ebp1* and *act*

Gene	Primers	Expected amplicon size
sqt	F: 5' TGCCGAGCACTCCAAGTATG 3'<br>R: 5' CATCAAGTTATCCAGGTGCC 3'	696 bp (un-spliced)<br>615 bp (spliced)
rnf180	F: 5' CTATTCCAAATGGCCTCTGC 3'<br>R: 5' CGTTTGTCCAGCAACAGAAA 3'	902 bp (un-spliced)<br>293 bp (spliced)
htr1ab	F: 5' CACCAAACCGACAATGTGAC 3'<br>R: 5' CAAGTTACCTGTCCAAGTGTCC 3'	269 bp
eif4ebp1	F: 5' AGTCAGGCAATTCCAACCAC 3'<br>R: 5' GGGGGCTGATGCTCTTATTA 3'	2801 bp (un-spliced)<br>286 bp (spliced)
act	F: 5' GGCTACAGCTTCACCACCA 3'<br>R: 5' TGCTGATCCACATCTGCTG 3'	657 bp (un-spliced)<br>487 bp (spliced)
